# Influence of Genetic Polymorphisms on the Age at Cancer Diagnosis in a Homogenous Lynch Syndrome Cohort of Individuals Carrying the *MLH1*:c.1528C>T South African Founder Variant

**DOI:** 10.3390/biomedicines12102201

**Published:** 2024-09-27

**Authors:** Lutricia Ndou, Ramadhani Chambuso, Ursula Algar, Paul Goldberg, Adam Boutall, Raj Ramesar

**Affiliations:** 1 UCT/MRC Genomic and Precision Medicine Research Unit, Division of Human Genetics, Department of Pathology, Institute of Infectious Disease and Molecular Medicine, University of Cape Town, and Affiliated Hospitals, Cape Town 7704, South Africa; ndxlut003@myuct.ac.za (L.N.); ramadhani.chambuso@uct.ac.za (R.C.); 2 The Colorectal Unit of the Department of Surgery, Groote Schuur Hospital, The University of Cape Town, Cape Town 7925, South Africa

**Keywords:** Lynch syndrome, age at cancer diagnosis, colorectal cancer, Lynch syndrome-associated cancer, extracolonic cancer, genetic polymorphisms, genetic modifiers, personalized screening

## Abstract

**Background:** High variability in the age at cancer diagnosis in Lynch syndrome (LS) patients is widely observed, even among relatives with the same germline pathogenic variant (PV) in the mismatch repair (MMR) genes. Genetic polymorphisms and lifestyle factors are thought to contribute to this variability. We investigated the influence of previously reported genetic polymorphisms on the age at cancer diagnosis in a homogenous LS cohort with a South African founder germline PV c.1528C>T in the *MLH1* gene. **Methods:** A total of 359 LS variant heterozygotes (LSVH) from 60 different families were genotyped for specific genetic polymorphisms in *GSTM1*, *GSTT1*, *CYP1A1*, *CYP17*, *PPP2R2B*, *KIF20A*, *TGFB1*, *XRCC5*, *TNF*, *BCL2*, *CHFR*, *CDC25C*, *ATM*, *TTC28*, *CDC25C*, *HFE*, *and hTERT* genes using Multiplex Polymerase Chain Reaction and MassArray methods. Kaplan–Meier survival analysis, univariate and multivariate Cox proportional hazards gamma shared frailty models adjusted for sex were used to estimate the association between age at cancer diagnosis and polymorphism genotypes. A *p*-value < 0.05 after correcting for multiple testing using the Benjamini–Hochberg method was considered significant at a 95% confidence interval. **Results:** We identified three genotypes in the cell-cycle regulation, DNA repair, and xenobiotic-metabolism genes significantly associated with age at cancer diagnosis in this cohort. The *CYP1A1* rs4646903 risk (GG) and *CDC25C* rs3734166 polymorphic (GA+AA) genotypes were significantly associated with an increased risk of a younger age at cancer diagnosis (Adj HR: 2.03 [1.01–4.08], *p* = 0.034 and Adj HR: 1.53 [1.09–2.14], *p* = 0.015, respectively). LSVH who were heterozygous for the *XRCC5* rs1051685 SNP showed significant protection against younger age at cancer diagnosis (Adj HR: 0.69 [CI, 0.48–0.99], *p* = 0.043). The risk of a younger age at any cancer diagnosis was significantly high in LS carriers of one to two risk genotypes (Adj HR: 1.49 [CI: 1.06–2.09], corrected *p* = 0.030), while having one to two protective genotypes significantly reduced the risk of developing any cancer and CRC at a younger age (Adj HR: 0.52 [CI: 0.37–0.73], and Adj HR: 0.51 [CI: 0.36–0.74], both corrected *p* < 0.001). **Conclusions:** Polymorphism genotypes in the cell-cycle regulation, DNA repair, and xenobiotic metabolizing genes may influence the age at cancer diagnosis in a homogenous LS cohort with a South African founder germline PV c.1528C>T in the *MLH1* gene.

## 1. Introduction

Lynch syndrome (LS) is an autosomal dominantly inherited Mendelian disorder associated with an increased risk of developing colorectal cancer (CRC) and other types of epithelial cancers at a younger age [1,2,3]. LS is caused by a germline pathogenic variant (PV) in one of the mismatch repair (MMR) genes (*MLH1*, *MSH2*, *MSH6*, and *PMS2*) or by epigenetic inactivation of the *MLH1* gene through hypermethylation of the CpG islands 5′ of this gene. In addition, structural mutations of the *EPCAM* gene, which is located approximately 15 kb upstream of the *MSH2* gene, may result in the inactivation of the latter gene, manifesting as LS [4,5,6,7,8]. Globally, LS accounts for approximately 3–5% of all CRC diagnoses, with a prevalence as high as 23% in patients diagnosed before the age of 35 years [3,9]. The risk of developing any cancer in LS patients differs widely depending on the mutated MMR gene and sex, with a lifetime risk of developing CRC being higher in *MLH1* and *MSH2* carriers than in *MSH6* and *PMS2* carriers and in men than in women [10,11,12,13,14,15,16]. In sub-Saharan Africa and South Africa, LS poses a significant health burden due to limited genetic screening and awareness, contributing to delayed diagnoses, poorer cancer surveillance, and poorer outcomes [17,18].

The biggest challenge with LS is the high variability in the age at cancer diagnosis, even in individuals carrying the same germline PV in the MMR genes. This is thought to be influenced by various environmental factors and genetic polymorphisms [16,19,20,21,22]. The latter are variations in the DNA sequence of a gene that can affect the phenotype expression of the primary PV [23,24]. Our research question seeks to investigate the influence of specific previously identified genetic polymorphisms on age at cancer diagnosis in LS variant heterozygotes (LSVH) [16,19,20,21,22]. If this research question is not addressed, LSVH will continue to receive generalized cancer surveillance and treatment plans, potentially resulting in delayed cancer detection, increased mortality, and reduced quality of life [21,25].

In our previous study using high-throughput HLA-allele typing in a homogenous cohort of LSVH with *MLH1*-associated germline PV, we showed that certain HLA allele variations may influence the age at cancer diagnosis [26]. We have also shown that the incidence of different solid cancers in LSVH in the same cohort can vary significantly by sex, even in individuals with the same founder germline PV in the *MLH1* gene [26]. This PV leads to a premature stop codon (p.Gln510Ter) in exon 13 of the *MLH1* gene, resulting in an absent or truncated protein product, which prevents *MLH1* from forming a functional heterodimer with *PMS2*. This defective MMR system leads to an accumulation of mutations, particularly in microsatellites, resulting in microsatellite instability (MSI), a characteristic feature of LS [20,27,28,29]. Polymorphisms that can influence the age at cancer diagnosis can be identified through genome-wide association studies and candidate gene studies focusing on specific genes thought to influence the condition based on prior knowledge, like in the present study [20,30,31,32,33,34,35,36]. Several polymorphisms in genes responsible for various biological pathways, such as cell cycle regulation [33,37], DNA repair [33], iron metabolism [36,38], telomerase maintenance [31,39], and xenobiotic metabolism [20,35,40,41,42], have been proposed to influence the age at cancer onset in LS. However, most of these studies were based on heterogeneous LS cohorts (i.e., without considering gene and PV specificity), which could have limited their ability to identify the actual effect of those genetic polymorphisms [25].

Polymorphisms in xenobiotic-metabolizing genes such as glutathione *S*-transferase group (GST) and cytochrome *P*450 group (CYP) have been shown to be capable of affecting the body’s ability to detoxify carcinogens, potentially accelerating cancer onset [43,44,45,46,47]. Similarly, variations in genes involved in cell cycle regulation [33,37,48,49], DNA repair [30,50,51,52], iron metabolism [36,53,54,55], and telomerase maintenance [39,56] can alter cellular processes, impacting the timing of cancer emergence [33,37,48,49,50,51,52]. We hypothesize that specific polymorphisms in cell cycle regulation, DNA repair, iron metabolism, telomerase maintenance, and xenobiotic metabolism genes may influence the age at cancer diagnosis in LSVH.

In this study, we investigated our hypothesis using a homogenous cohort of LSVH with a South African founder germline PV NM_000249.4(*MLH1*): c.1528C>T in the *MLH1* gene.

## 2. Materials and Methods

### 2.1. Patient Selection

**Figure 1:**
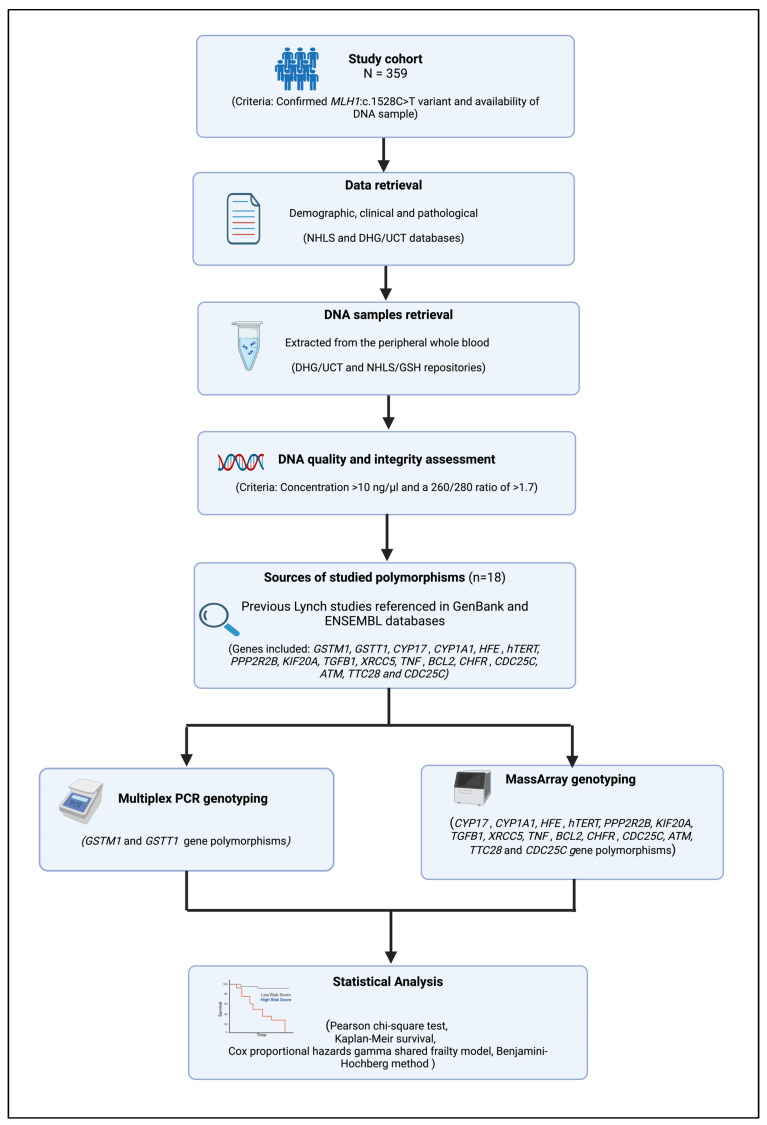
A flowchart summarizing the study methodology.

Over the past three decades, a total of 569 LSVH have been genetically diagnosed in the Division of Human Genetics (DHG) at the University of Cape Town (UCT) and the National Health Laboratory Services (NHLS) at Groote Schuur Hospital (GSH). These LSVH were recruited and referred from hospitals in the Western and Northern Cape provinces of South Africa with informed consent through genealogical tracing starting with a proband. The aim of recruiting these LSVH was to provide genetic surveillance and clinical management to them and their close relatives at high risk of developing hereditary cancers. Of these 569 LSVH, 426 from mixed-ancestry origin carry the same South African founder PV *MLH1*: c.1528C>T. As summarized in Figure 1, our study cohort included 359 of these LSVH from 60 different families, based on the following criteria:

- Confirmed genetic diagnosis of LS due to *MLH1*: c.1528C>T PV;
- Availability of a blood DNA sample with good quality and quantity according to the minimum requirements of the Multiplex PCR and MassArray genotyping assays (i.e., intact DNA with a concentration of >10 ng/µL and a 260/280 ratio of >1.7) [57].

### 2.2. Demographic and Pathological Data

Our LSVH cohort comprised 170 cancer-affected and 189 unaffected individuals. We extracted the pathological data of the cancer-affected LSVH from pathology reports obtained from the NHLS database. The demographic data of the cancer-affected and unaffected LSVH was retrieved from our in-house database at the DHG/UCT (Figure 1).

### 2.3. DNA Samples

We retrieved genomic DNA (gDNA) samples from the DHG/UCT and NHLS/GSH repositories. These samples were extracted from the peripheral whole blood of LSVH using the manual salting-out method at the DHG/UCT and the automated magnetic bead-based method (Maxwell@ 16, Promega, Madison, WI, USA) at the NHLS/GSH [58,59].

### 2.4. DNA Quality and Integrity Assessment

The concentration and purity (260/280 and 260/230 ratios) of the retrieved gDNA samples were assessed using the NanoDrop™ ND-1000 UV spectrophotometer (Thermo Fisher Scientific, Waltham, MA, USA), using 2 µL of a briefly vortexed gDNA sample. The gDNA integrity was analyzed by agarose gel electrophoresis using 1% (*w*/*v*) agarose gel consisting of agarose (UltraPure Agarose, Thermo Fisher Scientific, Waltham, MA, USA), 1X Tris-borate-EDTA (TBE) buffer and SYBR Safe Gel Stain (Invitrogen Corporation, Carlsbad, CA, USA). Each gDNA sample mixed with 3 μL of 6 X TriTrack DNA Loading Dye (Thermo Fisher Scientific, MA, USA) and 8 μL of GeneRuler 100 bp Plus DNA ladder (Thermo Fisher Scientific, USA) was loaded into the gel. The gel was run for 35 min at 120 Voltage, and gDNA bands were visualized and images captured using the UVIpro Gold Transilluminator (Uvitec, Cambridge, UK) (Supplementary Figure S1).

### 2.5. Sources of Studied Polymorphisms

A total of 18 polymorphisms previously identified by other authors as associated with cancer risk in LS and referenced in the GenBank (https://www.ncbi.nlm.nih.gov/genbank/, accessed on 1 November 2022) and ENSEMBL (https://www.ensembl.org/index.html, accessed on 1 November 2022) databases were included for examination in this study (Supplementary Table S1). These included the null polymorphism (entire gene deletion) of the *GSTM1* and *GSTT1* genes [20,41,60]. For the *CYP17* gene, we included the single nucleotide polymorphism (SNP) rs743572, while for the *CYP1A1* gene, the SNP rs4646903 and rs1048903 were included [35,40,42,52]. We also included the SNP rs1799945 in the *HFE* gene [36] and the SNP rs2075786 in the *hTERT* gene [39]. The SNPs in genes involved in cell cycle regulation and DNA repair included in this study are as follows: rs10477307 in the *PPP2R2B* gene, rs10038448 in the *KIF20A* gene, rs12980942 in the *TGFB1* gene, rs1051685 in the *XRCC5* gene, rs3093662 in the *TNF* gene, rs1531697 in the *BCL2* gene, rs11610954 in the *CHFR* gene, rs6874130 in the *CDC25C* gene, rs1800057 in the *ATM* gene, rs9608696 in the *TTC28* gene, rs6874130 and rs3734166 in the *CDC25C* gene [33].

### 2.6. Multiplex PCR

We examined the presence of the null genotype in both *GSTM1* and *GSTT1* genes using polymerase chain reaction and oligonucleotide sequences as previously described [20,61,62]. Primers were purchased from the Inqaba Biotech laboratory (Pretoria, South Africa) and diluted to a concentration of 20 μM from the 100 μM stock obtained by resuspending the lyophilized pellet in distilled water (dH20) for subsequent PCR work.

To determine the presence of *GSTM1* and *GSTT1* null genotypes, we included the β-globin gene primers as an internal control in a multiplex PCR reaction to document successful PCR amplification. PCR amplification was performed in a total volume of 25 µL containing ~100 ng of gDNA, 20 µM of each oligonucleotide (Supplementary Table S2) [20,61,62], 1X Green GoTaq reaction buffer (pH 8.5) (Promega, USA), 5 mM deoxyribonucleotide triphosphates (dNTPs) (Promega, USA), 25 mM magnesium chloride (MgCl2) (Promega, USA), 1X GoTaq Flexi DNA polymerase (Promega, USA). Reactions were subjected to a denaturation step of 95 °C for 4 min, followed by 30 cycles of 95 °C for 30 s, 50, 55, or 60 °C for 30 s, and 72 °C for 40 s, followed by a final extension step at 72 °C for 5 min [20]. The genotypes were ascertained on a 2% agarose gel by examining the gels for bands of the appropriate sizes as described by Felix et al. [20] (Supplementary Figure S2).

### 2.7. MassArray Genotyping

The Agena MassARRAY iPLEX genotyping assay (Agena Bioscience™, San Diego, CA, USA) was used for genotyping the other 15 selected SNPs in our cohort. SNP *CYP1A1* rs1048903 failed to be incorporated in the assay. The assay was performed at Inqaba Biotechnical Industries (Pretoria, South Africa) using the iPLEX assay protocol, which is based on single base extension or cleavage chemistry in conjunction with matrix-assisted laser desorption ionization–time of flight (MALDI-TOF) mass spectrometry as described by Ellis and Ong [57]. They were provided with 30 µL DNA from each of the selected LSVH at a concentration of >10 ng/µL. All except one of the 15 SNPs (specifically, rs960896) produced distinct genotype clusters in the iPLEX assay. Only samples with >80% coverage were included in the final analysis [57].

### 2.8. Statistical Analysis

Statistical analysis was performed using R (version 4.3.2). We used the Pearson chi-square test to assess the deviation from the expected Hardy–Weinberg equilibrium of each candidate polymorphism. To assess whether age at cancer diagnosis (used as a proxy for age of onset for cancer patients) differs by genotype, Kaplan–Meier survival curves stratified by genotypes were compared using the log-rank test. Thus, we defined first cancer as an outcome event of interest. All other patients’ data were censored at the time of last contact (for those who remained cancer-free) and at the time of death from other causes than cancer; the total risk analysis time for all patients included the period from the date of birth to the time of the first cancer event for cases or to the time of censoring for the remainder. The median age at diagnosis was defined as the age at which 50% of participants had been diagnosed with cancer. We explored the relationship between polymorphism genotype and age at cancer diagnosis (as a time-to-event outcome) using the Cox proportional hazards gamma shared frailty model to account for the relatedness of some of the LSVH. Two univariate models were fitted, a model containing polymorphism genotypes only and a model additionally adjusted for sex as a potential confounder. In addition, a multivariate model containing genotypes only and a model adjusted for sex as a potential confounder were also fitted. We obtained unadjusted and adjusted Hazard Ratios (HRs) and 95% confidence intervals (95% CI) for the effect of each candidate polymorphism genotype. In addition, we performed Kaplan–Meier and Cox proportional hazards gamma shared frailty analyses to determine the association between the aggregated number of likely risk and likely protective polymorphisms and age at cancer diagnosis. The significance level of univariate models and Kaplan–Meir analysis was set at *p*-value < 0.05 at 95% CI. Whereas, for multivariate models, the significance levels were considered when the *p*-value was <0.05 at a 95% CI after correcting for multiple comparisons using the Benjamini–Hochberg method to control for false discovery rate (FDR).

## 3. Results

### 3.1. Patient Cohort and Demographics

A total of one hundred and seventy (170) out of 359 LSVH were affected by cancer, with a mean age at cancer diagnosis of 44 (±10.9) years. There was a significant difference in the mean age at cancer diagnosis between male and female LSVH (*p* = 0.028, Table 1). The location of the tumor differs significantly between males and females, with a higher proportion of extra-colonic cancers in females (35.9%) than in males (6.5%) (*p* < 0.001). We also observed a higher proportion of poorly differentiated colorectal carcinomas in male patients than in female patients (23.3% vs. 6.0%, respectively, *p* = 0.009) (Table 1).

**Figure 2:**
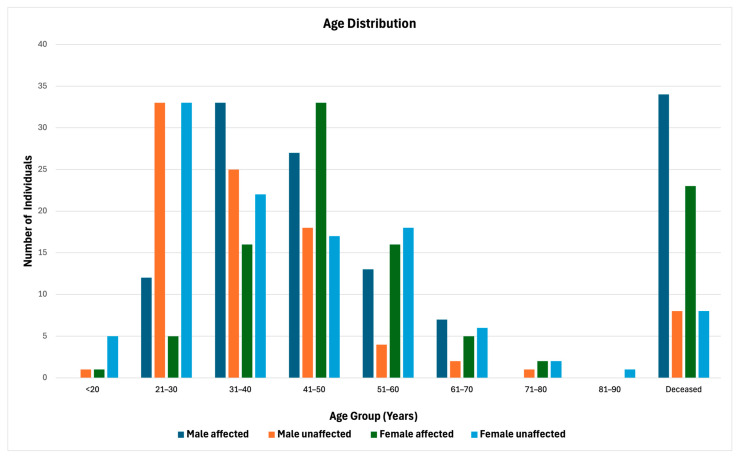
Age distribution of the LSVH in our study cohort, stratified by sex and health status (affected, unaffected, deceased).

To further illustrate the distribution of LSVH across different age groups in our cohort, we stratified the cohort by sex and health status (affected, unaffected, deceased), providing a comprehensive overview of demographic and health trends within this population (Figure 2). The 31–40 and 41–50 age groups show the highest counts of affected individuals in both males and females, respectively. A higher mortality rate is observed among affected males (Figure 2).

**Figure 3:**
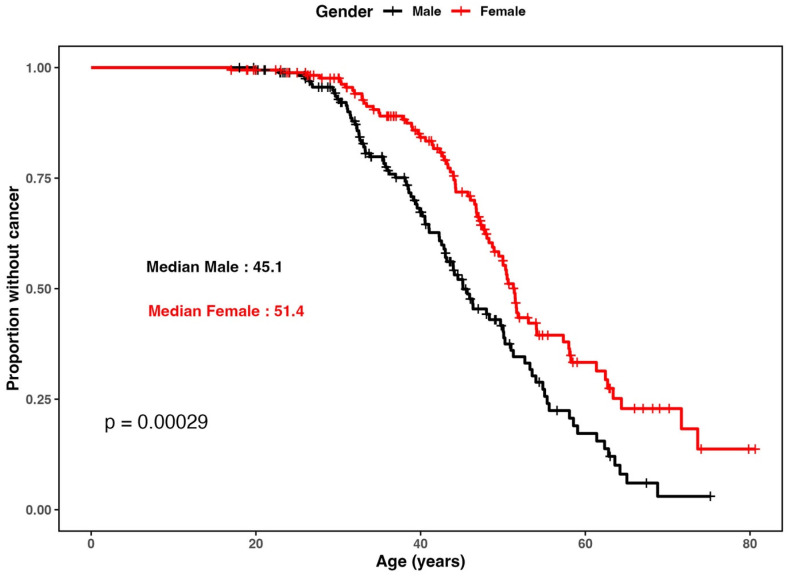
Kaplan–Meier survival plots by sex in the total cohort (i.e., the affected and unaffected LSVH). The curve shows the effect sex has on age at cancer (i.e., either CRC or extra-colonic cancer) diagnosis in LSVH. A significant difference in the age of cancer diagnosis can be seen between males (median age at diagnosis = 45 years) and females (median age at diagnosis = 51 years).

We observed a significant difference between the two Kaplan–Meier survival curves comparing the age at cancer diagnosis by sex in the total cohort of LSVH (comparing males and females; log-rank test *p* < 0.001) (Figure 3). Therefore, we adjusted all subsequent Cox regression analyses for sex to minimize the confounding impact of the strong sex bias. We excluded 19 samples from further analysis because the sample call rate after MassArray genotyping was <80%.

### 3.2. Single Nucleotide Polymorphisms Genotype Frequency Distribution and Hardy–Weinberg Equilibrium Analysis

We performed a Hardy–Weinberg equilibrium (HWE) analysis as a quality control measure of our genotyping data [63,64]. Only the *CHFR* rs11610954 SNP deviated from the expected allele frequencies under HWE (*p* = 0.024) (Supplementary Table S3).

### 3.3. Evaluation of the Impact of Polymorphism Genotypes on the Age at First Any-Cancer Diagnosis

We performed a Kaplan–Meier survival and Cox regression analysis to show the effect of polymorphism genotypes on the age at first any-cancer diagnosis (i.e., either CRC or extra-colonic cancer) in our LSVH cohort. We divided our results into two categories as follows:

i. Identified risk genotypes for age at first any cancer diagnosis.

LSVH with the *CYP1A1 Msp1 rs4646903* risk (GG) genotype had a 2-fold increased risk of younger age at cancer diagnosis than those with the wild-type (AA) genotype (Adj HR: 2.03 [1.01–4.08], *p* = 0.034) (Supplementary Table S4). A significant difference was also observed between Kaplan–Meier curves comparing the age at cancer diagnosis by *CYP1A1* rs4646903 SNP genotypes (comparing AA, AG, and GG; log-rank test *p* = 0.007). The median age at cancer diagnosis was 42 years for those with the heterozygous GG genotype compared to 48 years for those with the wild-type AA genotype (Supplementary Figure S3A). The *CDC25C* rs3734166 heterozygous (GA) genotype appeared to be associated with an increased risk of younger age at cancer diagnosis (Adj HR: 1.49 [CI: 1.05–2.11], *p* = 0.023) (Supplementary Table S4). However, the Kaplan–Meier survival analysis showed no significant difference in the age at cancer diagnosis when comparing GG, GA, and AA genotypes (log-rank test *p* = 0.035) (Supplementary Figure S3B). Further stratified analysis showed that having any polymorphic allele for the *CDC25C* rs3734166 (genotypes GA and AA) was associated with 1.5-times increased risk of younger age at cancer diagnosis compared to having a wild-type (GG) genotype (Adj HR: 1.53 [1.09–2.14], *p* = 0.015, log-rank test *p* = 0.110) (Supplementary Table S4).

ii. Identified protective genotypes for age at first any-cancer diagnosis.

LSVH with the *XRCC5* rs1051685 heterozygous (AG) genotype were less likely to develop cancer at a younger age than those with the wild-type (AA) genotype, with a 31% estimated reduction in risk (Adj HR: 0.69 [CI, 0.48–0.99], *p* = 0.043) (Supplementary Table S4). Kaplan–Meier survival analysis also showed a significant difference in age at cancer diagnosis between genotypes of *XRCC5* rs1051685 SNP (comparing AA, AG, and GG; log-rank test *p* = 0.040) (Supplementary Figure S3C). The median age at cancer diagnosis was 53 years for carriers of the heterozygous AG genotype, relative to 46 years for those with the wild-type AA genotype. The *GSTT1* null genotype appeared to reduce the risk of younger age at cancer diagnosis compared to the wild-type genotype (Adj HR: 0.65 [CI: 0.43–0.99], *p* = 0.044) (Supplementary Table S4). However, the Kaplan–Meier survival analysis showed no significant difference in the age at cancer diagnosis between carriers of *GSTT1* null genotype and wild-type genotype (Log-rank test *p* = 0.082) (Supplementary Figure S3D).

None of the other examined polymorphisms showed significant association with age at first any-cancer diagnosis in univariate analysis (*p* > 0.05), as shown in Supplementary Table S4. In addition, none of the 17 polymorphism genotypes in the multivariate model after correcting for multiple testing showed a significant association with age at any cancer diagnosis in our LS cohort (Supplementary Table S5).

### 3.4. Evaluation of the Impact of Polymorphism Genotype on the Age at First CRC Diagnosis

Considering that CRC is the most frequently diagnosed cancer in LS and the most observed cancer in this study cohort, we investigated whether polymorphism genotypes could impact the age at CRC diagnosis [65]. All LSVH affected with extra-colonic cancers (n = 34) were excluded from this analysis. We divided our results into two categories as follows:

i. Identified risk genotypes for age at CRC diagnosis.

The adjusted Cox regression analysis showed that LSVH with the *CDC25C* rs3734166 heterozygous (GA) genotype were more likely to develop CRC younger than those with the wild-type (GG) genotype (Adj HR: 1.50 [CI: 1.02–2.21], *p* = 0.039 and Adj HR: 1.55 [1.06–2.26], *p* = 0.022) (Supplementary Table S6). However, the Kaplan–Meier survival curves comparing the age at CRC diagnosis by genotype were not significantly different for the *CDC25C* rs3734166 SNP (comparing GG, GA, and AA; log-rank test *p* = 0.110) (Supplementary Figure S4A). In addition, the presence of any polymorphic allele for the *CDC25C* rs3734166 (genotypes GA and AA) was significantly associated with an elevated risk of young age at cancer diagnosis compared to having a wild-type (GG) genotype (Adj HR: 1.55 [1.06–2.26], *p* = 0.022; Log-rank test *p* = 0.035) (Supplementary Table S6).

ii. Identified protective genotypes for age at CRC diagnosis.

LSVH carriers of the *XRCC5* rs1051685 heterozygous (AG) genotype were more likely to be diagnosed with CRC at an older age than those with the wild-type (AA) genotype (39% estimated risk reduction; Adj HR: 0.61 [CI: 0.41–0.92], *p* = 0.019) (Supplementary Table S6). Kaplan–Meier survival curves comparing the age at CRC diagnosis between genotypes of the *XRCC5* rs1051685 SNP differed significantly (comparing AA, AG, and GG; log-rank test *p* = 0.040), with a 9-year difference in the median age at cancer diagnosis between heterozygous (AG) and wild-type (AA) genotype carriers (Supplementary Figure S4B). The *GSTT1* null genotype appeared to be associated with a 38% reduction in risk of young age at CRC diagnosis compared to the wild-type genotype (unadjusted HR: 0.62 [CI: 0.40–0.99], *p* = 0.044) (Supplementary Table S6). However, the Kaplan–Meier survival analysis showed no significant difference in the age at CRC diagnosis between the carriers of the *GSTT1* null genotype and wild-type genotype (log-rank test *p* = 0.130) (Supplementary Figure S4C).

No significant associations were observed with other examined polymorphisms and age at CRC diagnosis in univariate analysis (*p* > 0.05), as shown in Supplementary Table S6. Furthermore, the multivariate model with the 17 polymorphism genotypes showed no significant association with age at cancer diagnosis after correcting for multiple testing (Supplementary Table S7).

### 3.5. Aggregated Effect of Combined Risk Genotypes

We determined the aggregate effect of combined risk genotypes identified in univariate Cox regression analysis for any cancer (Supplementary Table S4) (*CYP1A1 Msp1* rs4646903 *GG* and *CDC25C* rs3734166 GA) on the age of cancer diagnosis in LS. Due to a low number of LSVH carrying two risk genotypes, we combined those carrying one and two genotypes into one group (one to two genotypes), similar to a published study [38]. LSVH with one to two risk genotypes were 1.5 times more at risk of developing any cancer at a younger age than those without risk genotypes (Adj HR: 1.49 [CI: 1.06–2.09], corrected *p* = 0.030; log-rank test: *p* = 0.024, Table 2 and Supplementary Figure S5A).

### 3.6. Aggregated Effect of Combined Protective Genotypes

Similarly, to determine the aggregated effect of combined identified protective genotypes identified in the univariate Cox regression analysis for any cancer and CRC (*XRCC5* rs1051685 AG, and *GSTT1* null genotypes, in Supplementary Tables S4 and S6) on age at cancer diagnosis in LS, we combined individuals carrying one to two protective genotypes into one group (one–two genotypes) [38]. Overall, having one to two protective genotypes was associated with a 48% and 49% reduction in risk of young age at any cancer and CRC diagnosis, respectively (Adj HR: 0.52 [CI: 0.37–0.73], corrected *p* < 0.001; LR test = 0.036, and HR: 0.51 [CI: 0.36–0.74], corrected *p* < 0.001; log-rank test < 0.001, respectively) (Table 3 and Supplementary Figure S5B,C).

## 4. Discussion

To our knowledge, this is the first large genetically homogeneous LS cohort study to examine the influence of genetic polymorphisms in cell cycle regulation, DNA repair, iron metabolism, telomerase maintenance, and xenobiotic metabolism genes on the age at cancer diagnosis. The study comprised 359 LSVH with a South African founder germline PV (*MLH1*: c.1528C>T). We identified specific risk and protective polymorphism genotypes significantly influencing the age of CRC and any cancer (i.e., either CRC or extra-colonic cancers) diagnosis in LS. The *CYP1A1 Msp1* rs4646903 *risk (GG)* genotype was significantly associated with an increased risk of younger age at any cancer diagnosis, while having any polymorphic allele for the *CDC25C* rs3734166 (genotypes GA and AA) was associated with an elevated risk of younger age at both CRC and any-cancer diagnosis in LS. The *XRCC5* rs1051685 heterozygous (AG) and *GSTT1* null genotypes conferred a protective effect that delayed age at cancer diagnosis in LS. Our results emphasize the potential for using identified polymorphism genotypes to personalize cancer surveillance and preventive strategies in LSVH.

The significant association of the *CYP1A1 Msp1* rs4646903 *GG* genotype with a younger age at any cancer diagnosis highlights the potential role of metabolic enzymes in modulating carcinogen activation and detoxification pathways, thereby accelerating tumorigenesis [66,67,68]. The effect of *CDC25C* rs3734166 polymorphic genotypes on age at cancer diagnosis implies that this SNP may affect *CDC25C* gene function by delaying cell cycle progression in response to stress, leading to earlier tumorigenesis in LSVH [69]. On the other hand, the potential protective effect observed with the *GSTT1* null genotype implies that carriers of the *GSTT1* null genotype could be less prone to the damaging effects of halogenated compounds via the GSTT1-1 pathway as previously hypothesized, ultimately delaying the onset of cancer [70,71]. Additionally, the effect of the *XRCC5* rs1051685 AG genotype implicates DNA repair fidelity and apoptosis regulation in cancer risk modulation, where efficient DNA repair and controlled cell death prevent malignant transformation [33,37,48,72,73,74]. Our findings reflect the intricate interplay between detoxification, cell-cycle regulation, DNA repair, and apoptosis in maintaining genomic stability and cancer prevention [75,76]. Understanding the influences of these genetic polymorphisms provides a deeper biological framework for developing targeted interventions, enhancing personalized medicine approaches in LS management, and ultimately improving patient prognoses by tailoring prevention and treatment strategies based on individual genetic profiles.

Our findings align with previous studies suggesting that the *CYP1A1* rs4646903 SNP elevates the risk of younger age at cancer diagnosis in LSVH [35,40]. However, one study reported that the risk of early CRC onset due to the *CYP1A1* rs4646903 SNP was only significantly increased in male patients, rather than the entire cohort [77]. This SNP is located at nucleotide 3801 of the *CYP1A1* gene in the 3′ flanking region, which alters the gene expression level [78,79]. We found that having any polymorphic allele for the *CDC25C* rs3734166 (genotypes GA and AA) increased the risk of young age at cancer diagnosis, which aligns with a previous finding [33]. The *CDC25C* rs3734166 is involved in the cell-cycle pathway; negative alterations in this gene could lead to dysregulation of the cell cycle, which is a crucial step in the initiation and development of cancer [80].

Several previous studies have reported contradicting findings on the effect of *GSTT1* null genotype on cancer risk in LS [20,35,60,81]. Felix et al. [20] and Moisio et al. [60] reported an increased risk of early-onset CRC in carriers of the *GSTT1* null genotype. On the other hand, two studies found no significant association between *GSTT1* null genotype and early onset of CRC in LS patients [35,81]. Our study findings contradict previous studies, as the *GSTT1* null genotype appeared to confer a protective effect against young age at cancer diagnosis. The protective effect of the *GSTT1* null genotype has been previously reported in bladder cancer [71]. However, our observation should be interpreted with caution, considering that the Kaplan–Meier survival analysis showed no significant difference in the age at cancer diagnosis between the genotypes of this polymorphism.

Interestingly, the *XRCC5* rs1051685 heterozygous AG genotype significantly conferred a protective effect against young age at cancer diagnosis, contradicting previously reported results [33]. Win et al., 2013 found no association between a polymorphism in the *XRCC5* gene and CRC risk in LS [30]. Further studies are needed to clarify the impact of *XRCC5* gene polymorphisms on cancer risk in LS. The *XRCC5* rs1051685 SNP has been associated with susceptibility to myeloma [82], hematologic toxicity in lung cancer patients treated with platinum-based chemotherapy [83], and the prognosis of non-metastasic lung cancer patients [84]. The discrepancies between our results and previously reported findings on the influence of *GSTT1* null and *XRCC5* rs1051685 SNP on the age at cancer diagnosis could be due to genetic heterogeneity among LS cohorts, different ethnicities, and varying sample sizes.

We found no significant associations between the other twelve polymorphisms (*HFE* rs1799945, *CYP17* rs743572, *hTERT* rs2075786, *PPP2R2B* rs10477307, *KIF20A* rs10038448, *TGFB1/CCDC97* rs12980942, *TNF* rs3093662, *CHFR* rs11610954, *CDC25C* rs6874130, *ATM* rs1800057, *TTC28* rs9608696, and *GSTM1* null) examined in our study and age at cancer diagnosis in univariate analyses. These polymorphisms were previously associated with cancer risk in LS [20,33,35,36,38,41,42,52,60,85]. The discrepancies with our current study findings could be due to the fact that these studies did not consider MMR gene specificity, except for our previous study [20]. Scott [25] recently highlighted the importance of considering gene specificity when elucidating the impact of genetic polymorphisms on age at cancer diagnosis in LS. Also, differences in ethnicities and sample sizes could explain the discrepancies observed. Considering that cancer is a multifactorial disease, it is crucial to explore the interactions between various polymorphisms and other covariates to gauge their influence on cancer development. In our study, after correcting for multiple testing in multivariate Cox regression analyses, none of the 17 polymorphism genotypes were significantly associated with age at cancer diagnosis. This could be explained by the use of a stringent correction method, which is necessary to control type I error but can decrease the likelihood of detecting true associations, especially when you have a larger number of covariates and a relatively small sample size. Future studies on genetically homogenous cohorts must ensure that the sample size and number of genetic polymorphisms are adequate to identify the true interactive effect of genetic polymorphisms on age at cancer diagnosis in LS.

Our study highlights how polymorphisms in genes beyond the primary MMR genes can contribute to the progression of cancer development in LS. This insight enhances our understanding of the multifactorial nature of cancer development, involving a complex interplay between primary pathogenic variants and secondary genetic polymorphisms. Understanding the genetic basis of the variability in age at cancer diagnosis in LSVH can inform personalized targeted preventive measures. For example, individuals with risk genetic polymorphisms might benefit from more frequent surveillance or specific lifestyle interventions to mitigate their elevated early cancer onset risk. Investigating these genetic polymorphisms in other genetically homogenous cohorts from different populations will help validate their roles and refine the existing predictive models for cancer onset risk in LS.

The main strengths of our study lie in the following: (i) We used a large genetically homogenous cohort of 359 mixed-ancestry LSVH with the South African founder germline PV (*MLH1*: c.1528C>T). We used this unique cohort to assess the influence of genetic polymorphisms on age at cancer diagnosis in this high cancer risk cohort. A genetically homogenous LS cohort reduces genetic variability among individuals, allowing for precise identification of the impact of other genetic polymorphisms on age at cancer diagnosis. (ii) We utilized Cox proportional hazards gamma shared frailty models to account for the relatedness of some LSVH, correcting for multiple testing using the Benjamini–Hochberg method and Hardy–Weinberg equilibrium analysis as a quality control measure for genotyping data to ensure methodological rigor and reliability of our findings. (iii) We made a comprehensive genetic analysis with a thorough evaluation of multiple genetic polymorphisms, covering key genes involved in cell regulation, detoxification, DNA repair, and iron metabolism.

The limitations include the following: (i) Potential population-specific effects may not be generalized to other ethnic groups of LSVH, which may limit the applicability of the results to diverse populations. (ii) The study’s cross-sectional nature limits the ability to infer cause-effect relationships between genetic polymorphisms and age at cancer diagnosis. (iii) There may be unmeasured confounders, such as lifestyle factors or environmental exposures. However, considering that most of these individuals originate from the same geographical location (the west coast of South Africa), we assumed that environmental factors did not influence the variability in age at cancer diagnosis observed in this cohort. (iv) Nineteen samples were excluded due to low genotyping call rates, which could introduce bias. (v) Only a selected number of genetic polymorphisms were analyzed, which may overlook other relevant yet unknown or understudied genetic polymorphisms impacting age at cancer diagnosis in LSVH.

Future studies may focus on addressing our limitations. However, despite these limitations, our study offers robust and impactful insights into the genetic polymorphisms that could influence the age at cancer diagnosis in a large, genetically homogenous cohort of LSVH. In contrast, we know that genetics alone does not explain the hereditability and the effect on the age of cancer onset. In addition to genetic polymorphisms, epigenetic modifications such as DNA methylation play a crucial role in cancer risk [86,87]. In LS, *MLH1* promoter hypermethylation is a well-established mechanism of gene silencing. Therefore, integrating genetic variants with epigenetic signatures could enhance the predictive power of risk models, offering a more nuanced and personalized approach to cancer prevention [88]. Furthermore, considering that LS is highly characterized by elevated immune responses due to persistent frameshift mutations that produce neoantigens, the immune system may potentially recognize and clear cancer cells [89,90,91]. The process of presenting neoantigens on the surface of the cell for recognition by immune cells is facilitated by the major histocompatibility complex proteins. Failure of this process can lead to immune evasion by cancer cells, which may facilitate cancer progression over time [90]. We, therefore, suggest that future studies should also focus on identifying epigenetic and immunogenic signatures associated with age at cancer diagnosis in LS.

The translational potential of our study is substantial, suggesting that genetic screening for identified risk and protective polymorphism genotypes could be integrated into cancer prediction models together with other epigenetic and immunogenic signatures to enhance personalized genetic targeted prevention and surveillance, thereby improving patient outcomes in LS. Our results emphasize the importance of genetically personalized prevention strategies, positioning our study as a valuable contribution to LS research from African populations.

## 5. Conclusions

We demonstrated that specific polymorphism genotypes in cell cycle, DNA repair, and xenobiotic metabolism genes may influence the age at cancer diagnosis in a genetically homogenous cohort of LSVH with a South African founder PV (*MLH1*: c.1528C>T). We have shown that having any polymorphic allele for the *CDC25C* rs3734166 (genotypes GA and AA) and *CYP1A1 Msp1* rs4646903 *GG risk* genotype may accelerate the onset of cancer in LSVH. Additionally, the heterozygous genotype for XRCC5 rs1051685 SNP appears to delay the onset of cancer in this population. Moreover, our findings indicate that the combined effect of risk genotypes increases the likelihood of younger age at cancer diagnosis, whereas having one to two protective genotypes reduces the risk of younger age at cancer diagnosis in LSVH. These findings highlight the translational potential of integrating genetic risk stratification of identified polymorphism genotypes into clinical practice, enabling genetic personalized surveillance and preventive strategies that can significantly improve patient outcomes and advance precision medicine in the management of LS.

## Figures and Tables

**Table 1:** Demographic and clinical characteristics of study participants stratified by sex.

Character	N (%) N = 359	Male (176)	Female (183)	*p*-Value
Status				
Cancer unaffected	189	84 (47.7%)	105 (57.4%)	0.085
Cancer affected	170	92 (52.3%)	78 (42.6%)	
Cancer-Affected				
Age at cancer diagnosis				
Mean (SD)	170	41.8 (10.9)	45.5 (10.6)	**0.028**
Median [Min, Max]	170	40.5 [19.9, 68.8]	46.6 [17.0, 73.6]	
Cancer type				
CRC	136	86 (93.5%)	50 (64.1%)	**<0.001**
Extra-colonic ^1^	34	6 (6.5%)	28 (35.9%)	
CRC histological grade				
Poorly differentiated	23	20 (23.3%)	3 (6.0%)	**0.009**
Moderately differentiated	113	66 (76.7%)	47 (94.0%)	
CRC histological type				
Adenocarcinoma	116	70 (81.4%)	46 (92.0%)	0.132
Mucinous adenocarcinoma	20	16 (18.6%)	4 (8.0%)	
Cancer recurrence				
Yes	36	15 (16.3%)	21 (26.9%)	0.134
No	134	77 (83.7%)	57 (73.1%)	

Note: ^1^ Extra-colonic cancers included gastric cancer, pancreatic cancer, cancer of the small intestine, endometrial cancer, ovarian cancer, bladder cancer, liver cancer, brain cancer, and skin cancer. Significant *p*-values are in bold.

**Table 2:** Aggregated Cox regression analysis for significant risk genotypes for any cancer in LSVH.

Number of Genotypes	Unadjusted HR (95% CI)	*p*-Value	# *p*-Value	* Adjusted HR (95% CI)	*p*-Value	# *p*-Value
No cancer risk genotype	Ref			Ref		
One to Two any cancer risk genotypes	1.44 (1.03–2.00)	**0.031**	0.050	1.49 (1.06–2.09)	**0.020**	**0.030**

* Adjusted for sex. # Corrected *p*-value using the Benjamini–Hochberg method. Note: Significant aggregated genotypes are in Bold. Abbreviations: HR. Hazards Ratio, CI. Confidence Interval.

**Table 3:** Aggregated Cox regression analysis for significant protective genotypes for any cancer and CRC in LSVH.

Number of Genotypes	Unadjusted HR (95% CI)	*p*-Value	# *p*-Value	* Adjusted HR (95% CI)	*p*-Value	# *p*-Value
No cancer risk genotype	Ref			Ref		
One to two any cancer protective genotypes	0.49 (0.35–0.69)	**<0.001**	**<0.001**	0.52 (0.37–0.73)	**<0.001**	**<0.001**
One to two CRC protective genotypes	0.50 (0.34–0.72)	**<0.001**	**<0.001**	0.51 (0.36–0.74)	**<0.001**	**<0.001**

* Adjusted for sex. # Corrected *p*-value using the Benjamini–Hochberg method. Note: Significant aggregated genotypes are in Bold. Abbreviations: HR. Hazards Ratio, CI. Confidence Interval.

## Data Availability

The data that support the findings of this study are available from the corresponding author upon reasonable request.

## Supplementary Materials

The following supporting information can be downloaded at: https://www.mdpi.com/article/10.3390/biomedicines12102201/s1: Table S1. List of genetic polymorphisms examined in the study; Table S2. Primer sequences for the PCR amplification of the GSTM1 and GSTT1 polymorphisms; Table S3. Genotype frequency distribution and HWE analysis for SNPs in LSVH cohort; Table S4. Comparison of Kaplan–Meier survival by genotype, univariate and sex-adjusted (as confounder) Cox regression analysis by genotype for any cancer; Table S5. Multivariate and sex-adjusted (as confounder) Cox regression analysis by genotype for any cancer; Table S6. Comparison of Kaplan–Meier survival by genotype, univariate and sex-adjusted (as confounder) Cox regression analysis by genotype for CRC; Table S7. Multivariate and sex-adjusted (as confounder) Cox regression analysis by genotype for CRC; Figure S1. DNA integrity assessment by agarose gel electrophoresis; Figure S2. Agarose gel electrophoresis showing the genotypes of *GSTM1* and *GSTT1* genes; Figure S3. Kaplan–Meir survival plots by *CYP1A1* rs4646903, *CDC25C* rs3734166, *XRCC5* rs1051685, *GSTT1*, *CDC25C* rs3734166 (GA+AA), and *XRCC5* rs1051685 (AG+GG) polymorphisms in LSVH; Figure S4. Kaplan–Meir survival plots by *CDC25C* rs3734166, *XRCC5* rs1051685, *GSTT1*, *CDC25C* rs3734166 (GA+AA), and *XRCC5* rs1051685 (AG+GG) polymorphisms in LSVH; Figure S5. Kaplan–Meier survival plots of age at cancer diagnosis by the number of risk and protective genotypes in LSVH.
