# Supplementary material for: Influence of Genetic Polymorphisms on the Age at Cancer Diagnosis in a Homogenous Lynch Syndrome Cohort of Individuals Carrying the MLH1:c.1528C>T South African Founder Variant
Source: Biomedicines. 2024 Sep 27;12(10):2201. doi: 10.3390/biomedicines12102201 (PMC11505229; doi:10.3390/biomedicines12102201)
Supplement: Supplementary file 1 [file biomedicines-12-02201-s001.zip › Supplementary Figure S1.pdf]

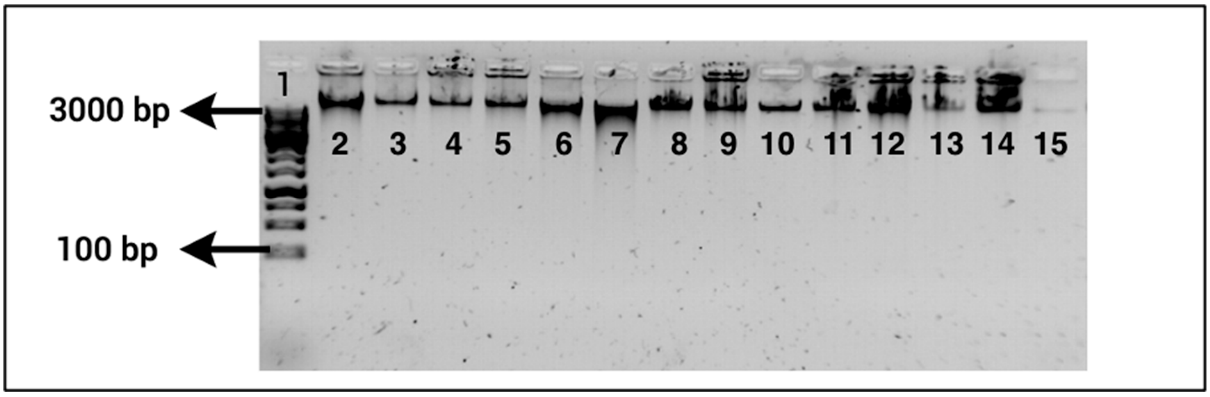

Supplementary Figure S1. DNA integrity assessment by agarose gel electrophoresis. The integrity of the retrieved gDNA samples was analysed by agarose gel electrophoresis. Three microlitres of each DNA sample was loaded onto a 1% agarose gel. Lane 1 shows a 100 bp Plus DNA ladder with a 100 bp to 3000 bp size range. Lanes 2 to 15 show intact retrieved gDNA samples, which were then included in the study. Lane 16 shows a degraded retrieved gDNA sample, which was excluded from the study. The image was captured using the UVIpro Gold Transilluminator gel documentation system.
