# Supplementary material for: Influence of Genetic Polymorphisms on the Age at Cancer Diagnosis in a Homogenous Lynch Syndrome Cohort of Individuals Carrying the MLH1:c.1528C>T South African Founder Variant
Source: Biomedicines. 2024 Sep 27;12(10):2201. doi: 10.3390/biomedicines12102201 (PMC11505229; doi:10.3390/biomedicines12102201)
Supplement: Supplementary file 1 [file biomedicines-12-02201-s001.zip › Supplementary Figure S2.pdf]

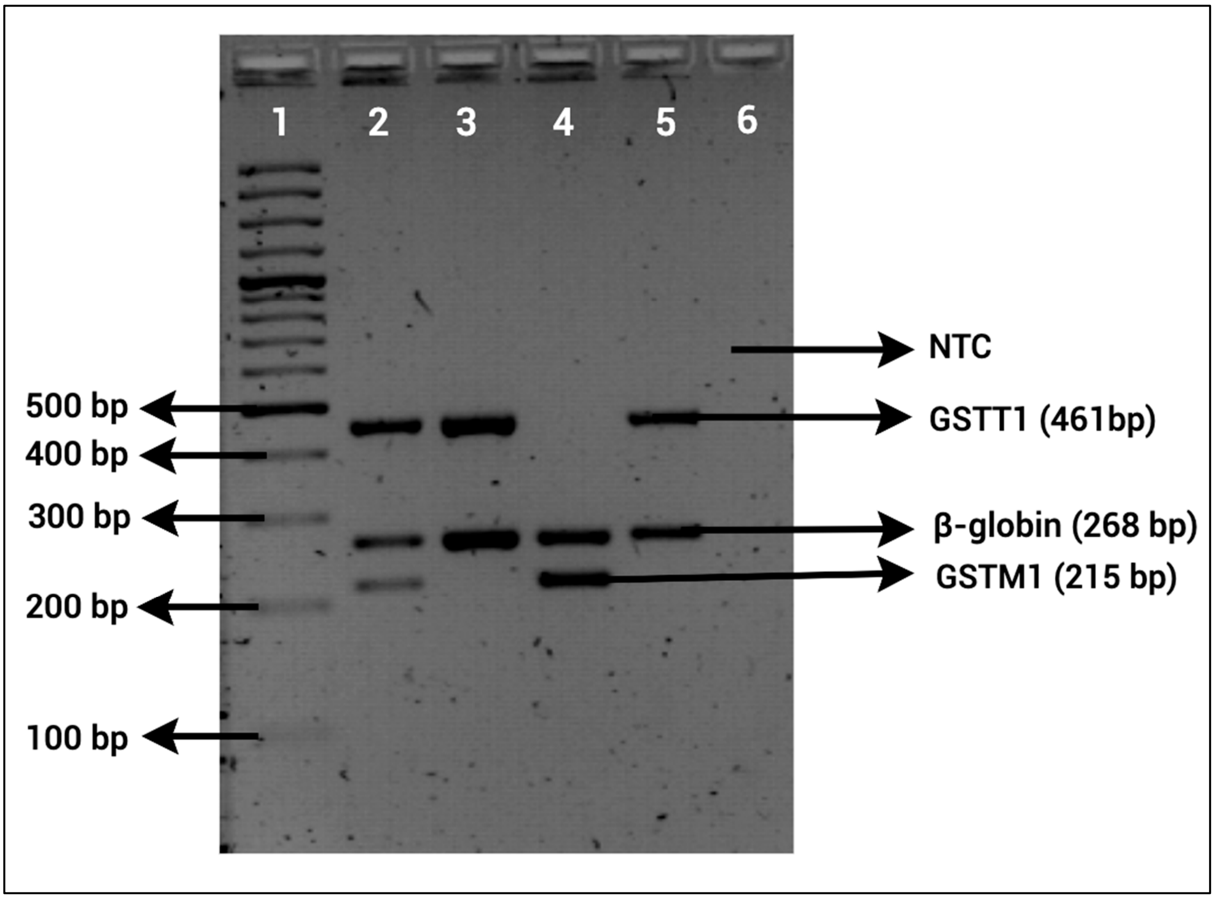

Supplementary Figure S2. Agarose gel electrophoresis showing the genotypes of *GSTM1* and *GSTT1* genes. The presence (wild-type) or absence (null genotype) of *GSTM1* and *GSTT1* genes was detected by the presence of a band at 215 bp (corresponding to *GSTM1*) and a band at 461 bp (corresponding to *GSTT1*). The bands at lane 1 indicate a molecular marker of 100 bp plus DNA ladder. A band at 268 bp (corresponding to the β-globin gene) was always present and was used as an internal control to document successful PCR amplification. Lane 2 indicates individuals with the presence of both *GSTM1* and *GSTT1* genotypes. Lanes 3 and 5 correspond to the individuals with *GSTM1* null and *GSTT1* present (461 bp) genotypes. Lane 4 represents the presence of the *GSTM*1 genotype and *GSTT1* null genotype. Lane 6 represents a no-template control.
