# Supplementary material for: Influence of Genetic Polymorphisms on the Age at Cancer Diagnosis in a Homogenous Lynch Syndrome Cohort of Individuals Carrying the MLH1:c.1528C>T South African Founder Variant
Source: Biomedicines. 2024 Sep 27;12(10):2201. doi: 10.3390/biomedicines12102201 (PMC11505229; doi:10.3390/biomedicines12102201)
Supplement: Supplementary file 1 [file biomedicines-12-02201-s001.zip › Supplementary Figure S3.pdf]

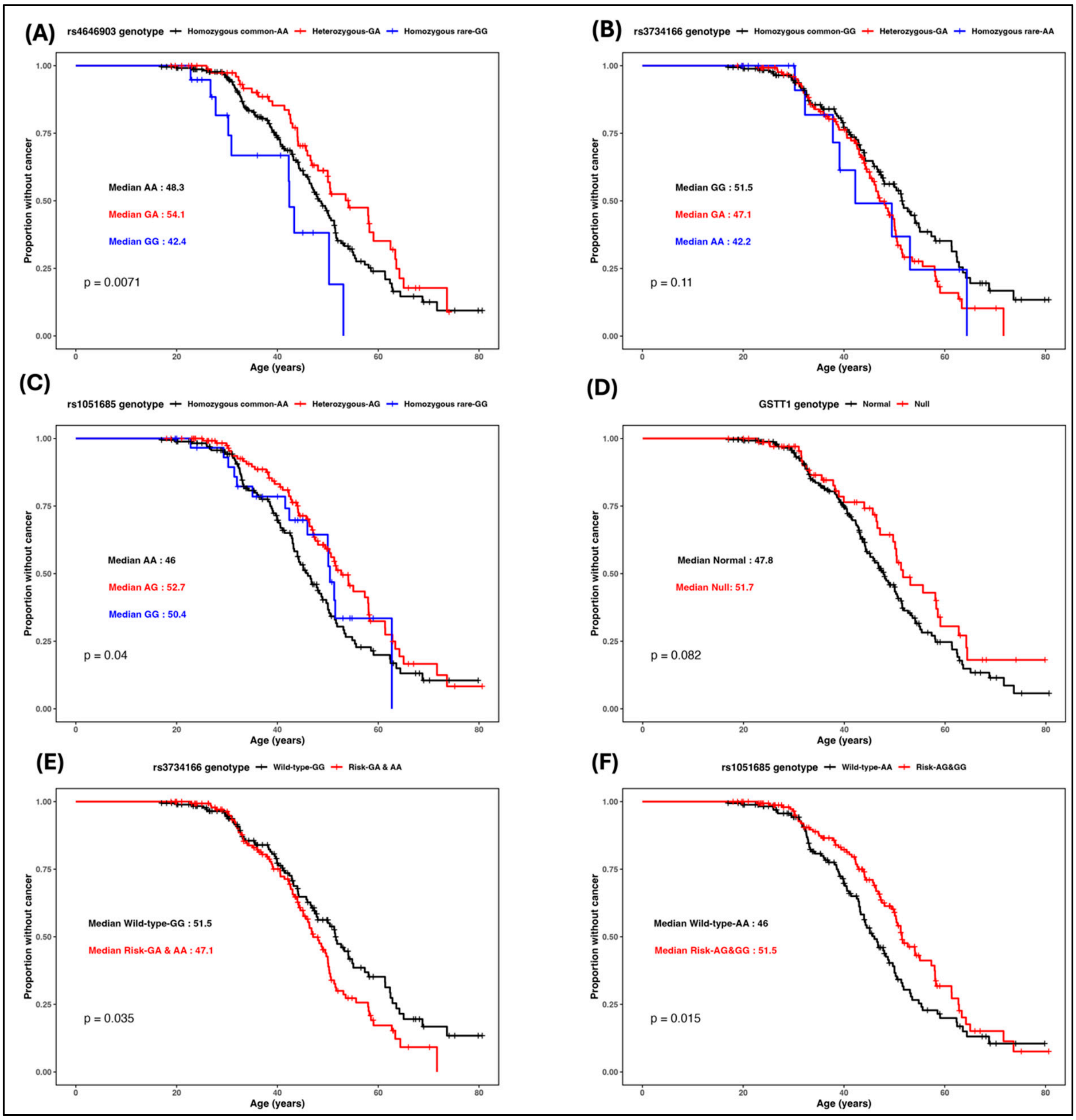

Supplementary Figure S3. Kaplan-Meir survival plots by *CYP1A1* rs4646903, *CDC25C* rs3734166, *XRCC5* rs1051685, *GSTT1*, *CDC25C* rs3734166 (GA+AA), and *XRCC5* rs1051685 (AG+GG) polymorphisms in Lynch syndrome variant heterozygotes (LSVH). These plots illustrate the effect of polymorphism genotypes on the age of LS cancer diagnosis in LSVH. (**A**) *CYP1A1* rs4646903 genotypes (AA, AG, and GG); (**B**) CDC25C rs3734166 (GG, GA, and AA); (**C**) *XRCC5* rs1051685 genotypes (AA, AG, and GG); (**D**) *GSTT1* genotypes (Normal and Null); (E) *CDC25C* rs3734166 Additive (GG, and GA+AA); (F) *XRCC5* rs1051685 genotypes (AA, and AG + GG)
