# Supplementary material for: Influence of Genetic Polymorphisms on the Age at Cancer Diagnosis in a Homogenous Lynch Syndrome Cohort of Individuals Carrying the MLH1:c.1528C>T South African Founder Variant
Source: Biomedicines. 2024 Sep 27;12(10):2201. doi: 10.3390/biomedicines12102201 (PMC11505229; doi:10.3390/biomedicines12102201)
Supplement: Supplementary file 1 [file biomedicines-12-02201-s001.zip › Supplementary Figure S4.pdf]

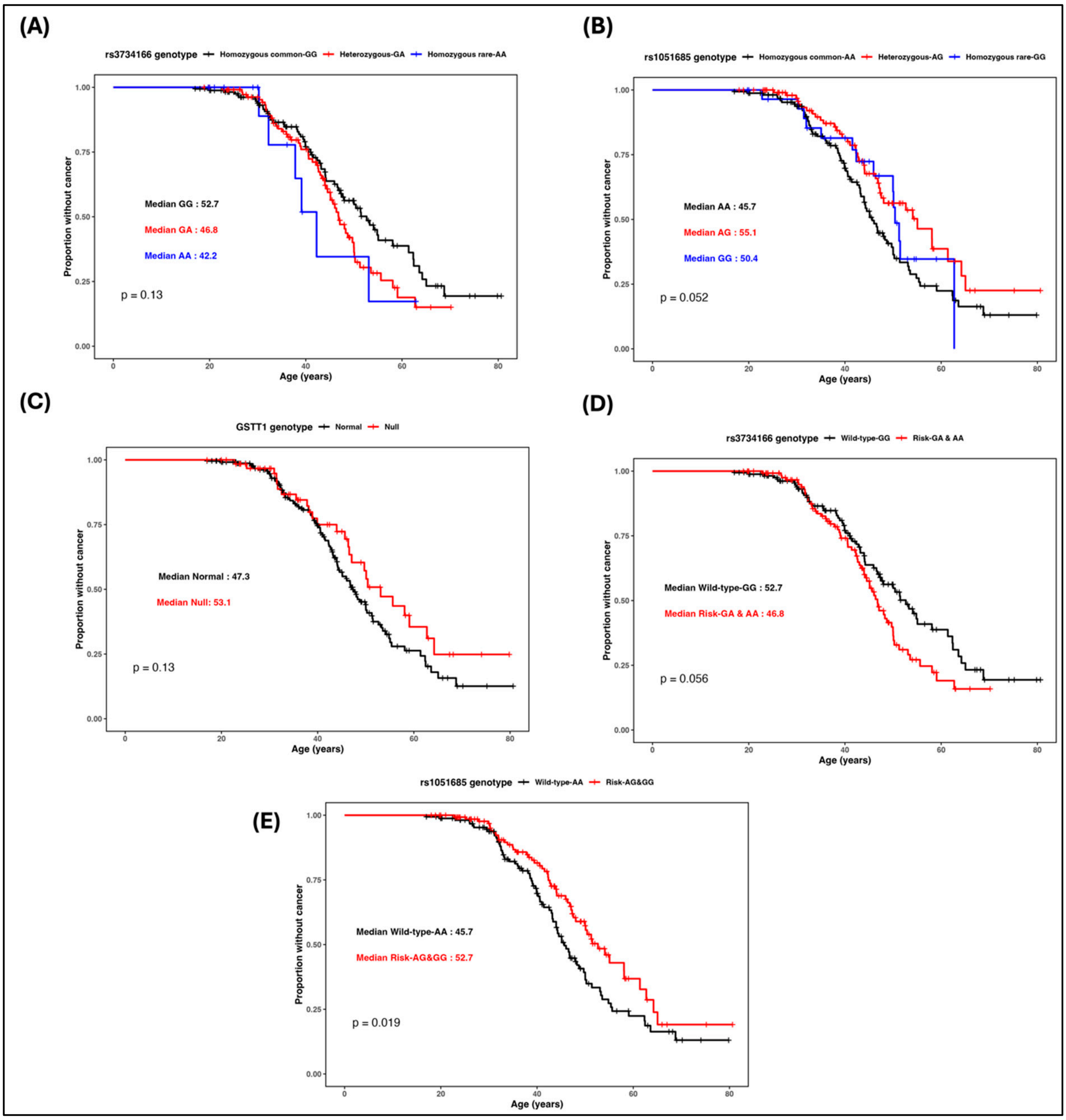

Supplementary Figure S4. Kaplan-Meir survival plots by *CDC25C* rs3734166, *XRCC5* rs1051685, *GSTT1*, *CDC25C* rs3734166 (GA+AA) and *XRCC5* rs1051685 (AG+GG) polymorphisms in LSVH. These plots illustrate the effect of polymorphism genotypes on the age of CRC diagnosis in LSVH. (**A**) *CDC25C* rs3734166 (GG, GA, and AA); (**B**) *XRCC5* rs1051685 genotypes (AA, AG, and GG); (**C**) *GSTT1* genotypes (Normal and Null); (**D**) CDC25C rs3734166 Additive (GG, and GA+AA); (**E**) *XRCC5* rs1051685 genotypes (AA, and AG + GG).
