# Supplementary material for: Influence of Genetic Polymorphisms on the Age at Cancer Diagnosis in a Homogenous Lynch Syndrome Cohort of Individuals Carrying the MLH1:c.1528C>T South African Founder Variant
Source: Biomedicines. 2024 Sep 27;12(10):2201. doi: 10.3390/biomedicines12102201 (PMC11505229; doi:10.3390/biomedicines12102201)
Supplement: Supplementary file 1 [file biomedicines-12-02201-s001.zip › Supplementary Figure S5.pdf]

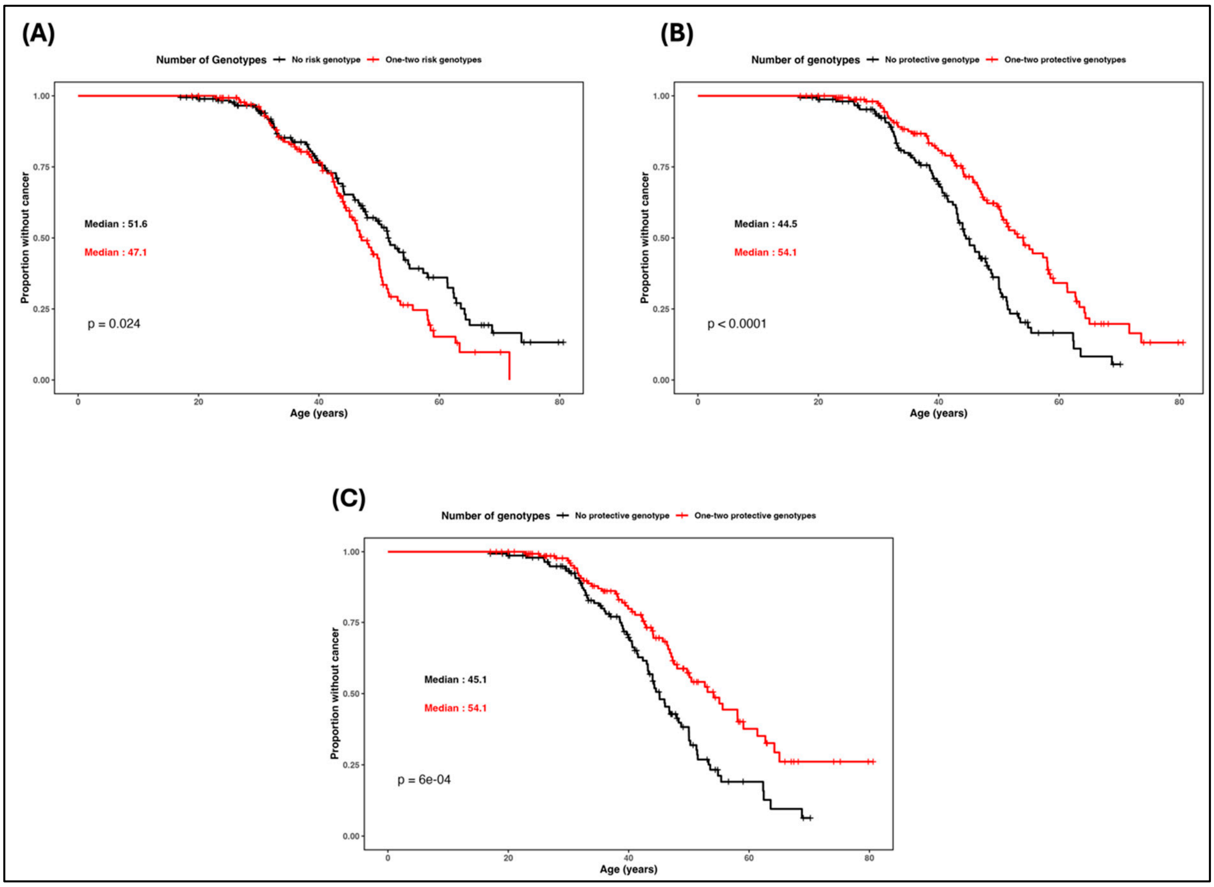

Supplementary Figure S5: Kaplan-Meier survival plots of age at cancer diagnosis by the number of risk and protective genotypes in LSVH. (**A**) The survival curves depict the survival to age at any cancer diagnosis for individuals with no risk genotype (black), and one to two risk genotypes (red). The difference in age at cancer diagnosis among the groups is statistically significant ($p = 0.024$). (**B**) The survival curves depict the survival to age at any cancer diagnosis for individuals with no protective genotype (black) and one to two protective genotypes (red). The difference in age at cancer diagnosis among the groups is statistically significant ($p < 0.001$). (**C**) A similar analysis is performed specifically for CRC protective genotypes, showing survival to age at CRC diagnosis for individuals with no protective genotype (black)and one to two protective genotypes (red), with a significant difference in age at cancer diagnosis ($p < 0.001$). Any cancer includes CRC, gastric cancer, pancreatic cancer, cancer of the small intestine, endometrial cancer, ovarian cancer, bladder cancer, liver cancer, brain cancer, and skin cancer.
