# Supplementary material for: Influence of Genetic Polymorphisms on the Age at Cancer Diagnosis in a Homogenous Lynch Syndrome Cohort of Individuals Carrying the MLH1:c.1528C>T South African Founder Variant
Source: Biomedicines. 2024 Sep 27;12(10):2201. doi: 10.3390/biomedicines12102201 (PMC11505229; doi:10.3390/biomedicines12102201)
Supplement: Supplementary file 1 [file biomedicines-12-02201-s001.zip › Supplementary Table S1.pdf]

Supplementary Table S1. List of genetic polymorphisms examined in the study.

**Polymorphism**	**Consequence type***	**Regulatory feature consequences***	**Regulatory Feature type**	**Source (studies DOI number)**
CYP1A1 I462V rs1048903	non coding transcript exon variant	Not available	Not available	https://doi.org/10.1158/1055-9965.Epi-08-0326<br><br>https://doi.org/10.1158/1055-9965.Epi-06-0040
CYP1A1 Msp1 rs4646903	intergenic variant	Not available	Gene expression	https://doi.org/10.1158/1055-9965.Epi-08-0326<br><br>https://doi.org/10.1158/1055-9965.Epi-06-0040<br><br>https://doi.org/10.1158/1078-0432.CCR-06-2987
CYP17 rs743572	5 prime UTR variant	Regulatory region variant	Enhancer	https://doi.org/10.1158/1078-0432.CCR-06-2987
hTERT rs2075786	intron variant	Not available	Not available	https://doi.org/10.1038/ejhg.2012.204
HFE H63D rs1799945	missense variant	Not available	Not available	https://doi.org/10.1002/ijc.24304
GSTM1	gene deletion	Not available	Lack of enzyme activity	https://doi.org/10.1016/j.mrfmmm.2006.09.004<br><br>https://doi.org/https://doi.org/10.3892/or.10.2.483<br><br>https://doi.org/10.1016/s0016-5085(98)70017-4
GSTT1	gene deletion	Not available	Lack of enzyme activity	https://doi.org/10.1016/j.mrfmmm.2006.09.004<br><br>https://doi.org/10.1016/s0016-5085(98)70017-4
TGFB1/CCDC97 rs12980942	intron variant	Regulatory region variant	Transcription Factor binding site	https://doi.org/10.1093/carcin/bgs344

PPP2R2B rs10477307	intron variant	Regulatory region variant	Enhancer	
KIF20A rs10038448	intron variant	Regulatory region variant	CTCF_binding_site	
CDC25C rs3734166	missense variant	Not available	Not available	
XRCC5 rs1051685	3 prime UTR variant	Regulatory region variant	Enhancer	
TNF rs3093662	intron variant	Not available	Not available	
BCL2 rs1531697	intron variant	Not available	Transcription Factor binding site	
TTC28 rs9608696	intron variant	Regulatory region variant	Enhancer	
CHFR rs11610954	intron variant	Not available	Not available	
CDC25C rs6874130	Transcription Factor binding site	Regulatory region variant	Enhancer	
ATM rs1800057	Missense variant	Not available	Not available	
