# Supplementary material for: Influence of Genetic Polymorphisms on the Age at Cancer Diagnosis in a Homogenous Lynch Syndrome Cohort of Individuals Carrying the MLH1:c.1528C>T South African Founder Variant
Source: Biomedicines. 2024 Sep 27;12(10):2201. doi: 10.3390/biomedicines12102201 (PMC11505229; doi:10.3390/biomedicines12102201)
Supplement: Supplementary file 1 [file biomedicines-12-02201-s001.zip › Supplementary Table S2.pdf]

Supplementary Table S2. Primer sequences for the PCR amplification of the GSTM1 and GSTT1 polymorphisms

Primer	Sequence	Size of PCR fragment (bp)
**GSTT1F**	TTCCTTACTGGTCCTCACATCTC	461
**GSTT1R**	TCACCGGATCATGGCCAGCA	
**GSTM1F**	GAA CTC CCT GAA AAG CTA AAG C	215
**GSTM1R**	GTT GGG CTC AAA TAT ACG GTG G	
**β-GlobinF**	CAA CTT CAT CCA CGT TCA CC	268
**β-GlobinR**	GAA GAG CCA AGG ACA GGT AC	
