# Supplementary material for: Influence of Genetic Polymorphisms on the Age at Cancer Diagnosis in a Homogenous Lynch Syndrome Cohort of Individuals Carrying the MLH1:c.1528C>T South African Founder Variant
Source: Biomedicines. 2024 Sep 27;12(10):2201. doi: 10.3390/biomedicines12102201 (PMC11505229; doi:10.3390/biomedicines12102201)
Supplement: Supplementary file 1 [file biomedicines-12-02201-s001.zip › Supplementary Table S3.pdf]

Supplementary Table S3. Genotype frequency distribution and HWE analysis for SNPs in LSVH cohort. Note: Significant SNP is in Bold. Abbreviations: SNP: Single nucleotide polymorphism, HWE: Hardy-Weinberg equilibrium (HWE)**.**

SNP	MAF (dbSNP Overall/African)	Observed genotype (N)	Cancer affected	Cancer unaffected	HWE
HFE H63D rs1799945	G=0.073/G=0.011				
CC		277	134	143	0.857
CG		56	26	30	
CT		2	1	1	
GG		2	1	1	
Missing		3	1	2	
CYP17 rs743572	G=0.411/G=0.326 & T=0.000				
AA		68	35	33	0.074
AG		156	81	75	
GG		60	24	36	
GT		23	13	10	
AT		32	10	22	
TT		1	0	1	
hTERT rs2075786	G=0.434/ G=0.421				
AA		108	48	60	0.250
GA		161	82	79	
GG		70	33	37	
Missing		1	0	1	
PPP2R2B rs10477307	A=0.274/A=0.490				
GG		129	70	59	0.128
GA		166	72	94	
AA		43	20	23	
Missing		2	1	1	
KIF20A rs10038448	C=0.668/C=0.679				
CC		213	99	114	0.680
GC		112	57	55	
GG		15	7	8	
TGFB1/CCDC97 rs12980942	A=0.133/ A=0.051				
GG		289	145	144	0.628
GA		46	15	31	
AA		4	3	1	
Missing		1	0	1	
XRCC5 rs1051685	G=0.172/G=0.334				
AA		176	90	86	0.852

AG		132	58	74	
GG		32	15	17	
TNF	G=0.080/G=				
rs3093662	0.077	273	136	137	0.723
AA		62	26	36	
AG		4	1	3	
GG		1	0	1	
Missing					
BCL2	A=0.289/A=				
rs1531697	0.208	168	76	92	0.355
TT		139	72	67	
TA		31	14	17	
AA		2	1	1	
Missing					
CHFR	T=0.059/				
rs11610954	T=0.000				
CC		291	137	154	**0.024**
CT		43	23	20	
TT		6	3	3	
CDC25C	C=0.378/				
rs6874130	C=0.478				
GG		104	48	56	0.162
GC		183	88	95	
CC		53	27	26	
ATM	G=0.009/				
rs1800057	G=0.0008	330	155	175	0.940
CC		10	8	2	
CG					
CYP1A1 Msp1	G=0.293/				
rs4646903	G=0.235				
AA		230	112	118	0.100
AG		85	40	45	
GG		19	10	9	
Missing		6	1	5	
TTC28	*G=0.000031/				
rs9608696	G=0.000				
TT		329	156	173	NA
GG		0	0	0	
Missing		11	7	4	
CDC25C	A=0.390/				
rs3734166	A=0.477				
GG		185	77	108	0.244
GA		133	76	57	
AA		20	8	12	
Missing		2	2	0	

***Allele frequency obtain from gnomAD database, the rest are from the 1000 Genomes.**
