# Supplementary material for: Influence of Genetic Polymorphisms on the Age at Cancer Diagnosis in a Homogenous Lynch Syndrome Cohort of Individuals Carrying the MLH1:c.1528C>T South African Founder Variant
Source: Biomedicines. 2024 Sep 27;12(10):2201. doi: 10.3390/biomedicines12102201 (PMC11505229; doi:10.3390/biomedicines12102201)
Supplement: Supplementary file 1 [file biomedicines-12-02201-s001.zip › Supplementary Table S4.pdf]

**Supplementary Table S4.** Comparison of Kaplan-Meier survival by genotype, univariate and sex-adjusted (as confounder) Cox regression analysis by genotype for any cancer. Note: Significant polymorphism genotypes are in Bold. Abbreviations: HR: Hazards Ratio, CI: Confidence Interval, Ref: Reference genotype.

| Polymorphism | Genotype (N) | Cancer affected (N) | Log-rank test P | Univariate HR (95% CI) | P-value | *Adjusted HR (95% CI) | P-value |
|---|---|---|---|---|---|---|---|
| HFE H63D rs1799945 | | | | | | | |
| CC | 277 | 134 | 0.900 | Ref | | Ref | |
| CG | 56 | 26 | | 0.83 (0.51-1.34) | 0.450 | 0.89 (0.55-1.46) | 0.660 |
| CT | 2 | 1 | | 0.71 (0.10-5.19) | 0.730 | 0.50 (0.07-3.70) | 0.500 |
| GG | 2 | 1 | | 0.46 (0.06-3.42) | 0.450 | 0.39 (0.05-2.86) | 0.350 |
| Any G/T (CG+CT+GG) | 58 | 27 | 0.890 | 0.80 (0.50-1.30) | 0.380 | 0.86 (0.52-1.39) | 0.530 |
| CYP17 rs743572 | | | | | | | |
| AA | 68 | 35 | 0.380 | Ref | | Ref | |
| AG | 156 | 81 | | 1.13 (0.74-1.71) | 0.570 | 1.09 (0.72-1.67) | 0.680 |
| GG | 60 | 24 | | 0.92 (0.53-1.69) | 0.780 | 0.93 (0.53-1.65) | 0.830 |
| GT | 23 | 13 | | 1.32 (0.66-2.64) | 0.440 | 1.47 (0.72-2.98) | 0.290 |
| AT | 32 | 10 | | 0.55 (0.27-1.14) | 0.110 | 0.52 (0.25-1.08) | 0.082 |
| TT | 1 | 0 | | 0.00 (0.00-inf) | 1.000 | 0.00 (0.00-inf) | 1.000 |
| Any G/T (AG+GG+GT+AT+TT) | 272 | 128 | 0.760 | 1.02 (0.69-1.51) | 0.940 | 1.00 (0.67-1.49) | 1.000 |
| hTERT rs2075786 | | | | | | | |
| AA | 108 | 48 | 0.570 | Ref | | Ref | |
| AG | 161 | 86 | | 1.35 (0.92-1.99) | 0.130 | 1.32 (0.90-1.98) | 0.150 |
| GG | 70 | 33 | | 1.54 (0.95-2.48) | 0.079 | 1.46 (0.91-2.37) | 0.120 |
| Any G (AG+GG) | 231 | 115 | 0.300 | 1.39 (0.96-2.01) | 0.080 | 1.36 (0.94-1.96) | 0.100 |
| PPP2R2B rs10477307 | | | | | | | |
| GG | 129 | 70 | 0.280 | Ref | | Ref | |
| GA | 166 | 72 | | 0.77 (0.55-1.08) | 0.130 | 0.75 (0.53-1.05) | 0.096 |
| AA | 43 | 20 | | 0.83 (0.49-1.42) | 0.500 | 0.85 (0.49-1.47) | 0.560 |
| Any A(GA+AA) | 209 | 92 | 0.110 | 0.78 (0.57-1.08) | 0.140 | 0.77 (0.55-1.06) | 0.110 |
| KIF20A rs10038448 | | | | | | | |
| CC | 213 | 99 | 0.480 | Ref | | Ref | |
| CG | 112 | 57 | | 1.22 (0.86-1.72) | 0.270 | 1.33 (0.94-1.90) | 0.110 |
| GG | 15 | 7 | | 1.46 (0.65-3.27) | 0.350 | 1.63 (0.73-3.67) | 0.240 |
| Any G (CG+GG) | 127 | 64 | 0.240 | 1.24 (0.88-1.73) | 0.220 | 1.36 (0.96-1.92) | 0.080 |
| TGFB1/CCDC97 rs12980942 | | | | | | | |
| GG | 289 | 145 | 0.290 | Ref | | Ref | |
| GA | 46 | 15 | | 0.68 (0.39-1.20) | 0.190 | 0.66 (0.37-1.18) | 0.160 |

| | | | | | | | |
|---|---|---|---|---|---|---|---|
| AA | 4 | 3 | | 1.57 ( 0.44-5.28) | 0.500 | 1.13 (0.32-4.03) | 0.850 |
| Any A (GA+AA) | 50 | 18 | 0.180 | 0.75 (0.44-1.27) | 0.280 | 0.71 (0.41-1.21) | 0.210 |
| XRCC5 rs1051685 | | | | | | | |
| AA | 176 | 90 | **0.040** | Ref | | Ref | |
| AG | 132 | 58 | | 0.65 (0.46-0.92) | **0.016** | 0.69 (0.48-0.99) | **0.043** |
| GG | 32 | 15 | | 0.85 (0.48-1.51) | 0.570 | 0.94 (0.56-1.69) | 0.850 |
| Any G (AG+GG) | 164 | 73 | **0.015** | 0.68 (0.49-0.95) | **0.023** | 0.73 (0.52-1.02) | 0.068 |
| TNF rs3093662 | | | | | | | |
| AA | 273 | 136 | 0.260 | Ref | | Ref | |
| AG | 62 | 26 | | 1.22 (0.76-1.98) | 0.410 | 1.11 (0.68-1.80) | 0.670 |
| GG | 4 | 1 | | 0.15 (0.02-1.10) | 0.062 | 0.16 (0.02-1.20) | 0.075 |
| Any G (AG+GG) | 66 | 27 | 0.810 | 0.97 (0.61-1.52) | 0.880 | 0.90 (0.57-1.44) | 0.670 |
| BCL2 rs1531697 | | | | | | | |
| TT | 168 | 76 | 0.560 | Ref | | Ref | |
| TA | 139 | 72 | | 1.12 (0.80-1.56) | 0.510 | 1.10 (0.78-1.53) | 0.590 |
| AA | 31 | 14 | | 1.00 (0.54-1.87) | 0.999 | 0.80 (0.42-1.52) | 0.490 |
| Any A (TA+AA) | 170 | 86 | 0.660 | 1.10 (0.80-1.52) | 0.560 | 1.05 (0.76-1.46) | 0.770 |
| CHFR rs11610954 | | | | | | | |
| CC | 291 | 137 | 0.270 | Ref | | Ref | |
| CT | 43 | 23 | | 1.46 (0.90-2.37) | 0.120 | 1.41 (0.87-2.32) | 0.160 |
| TT | 6 | 3 | | 1.24 (0.38-4.04) | 0.720 | 1.11 (0.34-3.63) | 0.860 |
| Any T (CT+TT) | 49 | 26 | 0.110 | 1.44 (0.90-2.29) | 0.130 | 1.38 (0.86-2.21) | 0.180 |
| CDC25C rs6874130 | | | | | | | |
| GG | 104 | 48 | 0.880 | Ref | | Ref | |
| GC | 183 | 88 | | 1.05 (0.73-1.51) | 0.780 | 1.11 (0.77-1.61) | 0.580 |
| CC | 53 | 27 | | 1.01 (0.62-1.65) | 0.970 | 1.20 (0.72-1.99) | 0.480 |
| Any C(GC+CC) | 236 | 115 | 0.620 | 1.04 (0.74-1.48) | 0.810 | 1.13 (0.79-1.61) | 0.520 |
| ATM | | | | | | | |
| rs1800057 | 330 | 155 | 0.520 | Ref | | Ref | |
| CC | 10 | 8 | | 1.44 (0.62-3.31) | 0.390 | 1.47 (0.63-3.49) | 0.370 |
| CG | | | | | | | |
| CYP1A1 Msp1 rs4646903 | | | | | | | |
| AA | 230 | 112 | **0.007** | Ref | | Ref | |
| AG | 85 | 40 | | 0.68 (0.46-1.00) | 0.053 | 0.68 (0.46-1.00) | 0.051 |
| GG | 19 | 10 | | 2.05 (1.02-4.11) | **0.043** | 2.03 (1.01-4.08) | **0.047** |
| Any G (AG+GG) | 104 | 50 | 0.240 | 0.79 (0.55-1.13) | 0.200 | 0.79 (0.55-1.13) | 0.200 |

| | | | | | | | | |
|---|---|---|---|---|---|---|---|---|
| TTC28 | | | | | | | | |
| rs9608696 | 329 | 156 | - | - | - | - | - | |
| TT | 0 | 0 | | | | | | |
| GG | | | | | | | | |
| CDC25C | | | | | | | | |
| rs3734166 | | | | | | | | |
| GG | 185 | 77 | 0.110 | Ref | | Ref | | |
| GA | 133 | 76 | | 1.41 (1.01-1.98) | **0.045** | 1.49(1.05- 2.11) | **0.023** | |
| AA | 20 | 8 | | 1.53 (0.72-3.28) | 0.270 | 1.99 (0.92-4.31) | 0.081 | |
| Any A (GA+AA) | 153 | 84 | **0.035** | 1.42 (1.02-1.98) | **0.037** | 1.53 (1.09-2.14) | **0.015** | |
| GSTM1 | | | | | | | | |
| + (Present) | 275 | 129 | 0.260 | Ref | | Ref | | |
| - (Null) | 55 | 30 | | 1.41(0.90- 2.21) | 0.130 | 1.36(0.86- 2.16) | 0.190 | |
| GSTT1 | | | | | | | | |
| + (Present) | 256 | 126 | 0.082 | Ref | | Ref | | |
| - (Null) | 74 | 33 | | 0.65 (0.43-0.97) | **0.036** | 0.65(0.43- 0.99) | **0.044** | |

*Adjusted for sex
