# Supplementary material for: Influence of Genetic Polymorphisms on the Age at Cancer Diagnosis in a Homogenous Lynch Syndrome Cohort of Individuals Carrying the MLH1:c.1528C>T South African Founder Variant
Source: Biomedicines. 2024 Sep 27;12(10):2201. doi: 10.3390/biomedicines12102201 (PMC11505229; doi:10.3390/biomedicines12102201)
Supplement: Supplementary file 1 [file biomedicines-12-02201-s001.zip › Supplementary Table S5.pdf]

Supplementary Table S5. Multivariate and sex-adjusted (as confounder) Cox regression analysis by genotype for any cancer. Note: Significant polymorphism genotypes are in Bold. Abbreviations: HR: Hazards Ratio, CI: Confidence Interval, Ref: Reference genotype.

Polymorphism	Genotype (N)	Cancer affected (N)	HR (95% CI)	P-value	#P-value	*Adjusted HR (95% CI)	P-value	#P-value
HFE H63D rs1799945								
CC	277	134	Ref			Ref		
CG	56	26	0.96 (0.58-1.59)	0.870	0.982	1.03 (0.62-1.73)	0.900	0.983
CT	2	1	1.20 (0.15-9.61)	0.870	0.982	0.85 (0.11-6.78)	0.880	0.983
GG	2	1	0.36 (0.05-2.83)	0.330	0.600	0.27 (0.03-2.19)	0.220	0.513
CYP17 rs743572								
AA	68	35	Ref			Ref		
AG	156	81	1.53 (0.95-2.46)	0.080	0.342	1.56 (0.96-2.53)	0.070	0.267
GG	60	24	1.01 (0.56-1.82)	0.980	0.999	1.00 (0.55-1.80)	1.000	0.999
GT	23	13	1.83 (0.87-3.84)	0.110	0.346	2.01 (0.94-4.32)	0.072	0.267
AT	32	10	0.48 (0.21-1.09)	0.080	0.342	0.48 (0.21-1.08)	0.076	0.267
TT	1	0	0.00 (0.00-inf)	1.000	0.999	0.00 (0.00-inf)	1.000	0.999
hTERT rs2075786								
AA	108	48	Ref			Ref		
AG	161	86	1.20 (0.80-1.78)	0.380	0.634	1.21 (0.80-1.82)	0.360	0.631
GG	70	33	1.39 (0.85-2.29)	0.190	0.494	1.44 (0.87-2.37)	0.160	0.419
PPP2R2B rs10477307								
GG	129	70	Ref			Ref		
GA	166	72	0.73 (0.50-1.07)	0.110	0.345	0.71 (0.48-1.05)	0.088	0.279
AA	43	20	0.74 (0.40-1.36)	0.330	0.600	0.76 (0.41-1.40)	0.380	0.631
KIF20A rs10038448								
CC	213	99	Ref			Ref		
GC	112	57	0.85 (0.48-1.49)	0.560	0.737	0.92 (0.51-1.65)	0.780	0.959
GG	15	7	1.09 (0.28-4.22)	0.900	0.989	0.95 (0.25-3.53)	0.940	0.993
TGFB1/CCDC97 rs12980942								
GG	289	145	Ref			Ref		
GA	46	15	0.60 (0.33-1.09)	0.094	0.346	0.57 (0.31-1.05)	0.071	0.267
AA	4	3	2.92(0.72-11.77)	0.130	0.376	2.22 (0.56-8.91)	0.260	0.530
XRCC5 rs1051685								
AA	176	90	Ref			Ref		

AG<br>GG	132<br>32	58<br>15	0.58 (0.40-0.84)<br>0.79 (0.42-1.48)	**0.005**<br>0.460	0.157<br>0.676	0.62 (0.42-0.92)<br>0.90 (0.48-1.68)	**0.017**<br>0.730	0.267<br>0.947
TNF rs3093662<br>AA<br>AG<br>GG	 273<br>62<br>4	 136<br>26<br>1	 Ref<br>1.35 (0.82-2.23)<br>0.41 (0.05-3.19)	 <br>0.240<br>0.390	 <br>0.522<br>0.634	 Ref<br>1.17 (0.70-1.95)<br>0.36 (0.05-2.78)	 <br>0.550<br>0.330	 <br>0.797<br>0.630
BCL2<br>rs1531697<br>TT<br>TA<br>AA	 <br>168<br>139<br>31	 <br>76<br>72<br>14	 <br>Ref<br>1.10 (0.76-1.59)<br>0.53 (0.26-1.07)	 <br><br>0.610<br>0.078	 <br><br>0.763<br>0.342	 <br>Ref<br>1.10 (0.76-1.59)<br>0.44 (0.22-0.90)	 <br><br>0.620<br>**0.024**	 <br><br>0.830<br>0.267
CHFR<br>rs11610954<br>CC<br>CT<br>TT	 <br>291<br>43<br>6	 <br>137<br>23<br>3	 <br>Ref<br>1.76 (1.04-2.99)<br>1.67 (0.49-5.65)	 <br><br>**0.037**<br>0.410	 <br><br>0.333<br>0.639	 <br>Ref<br>1.73 (1.00-2.98)<br>1.53 (0.46-5.18)	 <br><br>**0.048**<br>0.490	 <br><br>0.267<br>0.780
CDC25C<br>rs6874130<br>GG<br>GC<br>CC	 <br>104<br>183<br>53	 <br>48<br>88<br>27	 <br>Ref<br>1.02 (0.65-1.59)<br>0.86 (0.44-1.67)	 <br><br>0.940<br>0.650	 <br><br>0.998<br>0.787	 <br>Ref<br>0.94 (0.60-1.47)<br>0.84 (0.43-1.63)	 <br><br>0.790<br>0.600	 <br><br>0.959<br>0.830
ATM<br>rs1800057<br>CC<br>CG	 <br>330<br>10	 <br>155<br>8	 <br>Ref<br>1.33 (0.55-3.26)	 <br><br>0.530	 <br><br>0.716	 <br>Ref<br>1.08 (0.44-2.64)	 <br><br>0.870	 <br><br>0.983
CYP1A1 Msp1<br>rs4646903<br>AA<br>AG<br>GG	 <br>230<br>85<br>19	 <br>112<br>40<br>10	 <br>Ref<br>0.64 (0.42-0.98)<br>1.47 (0.67-3.22)	 <br><br>**0.039**<br>0.340	 <br><br>0.333<br>0.600	 <br>Ref<br>0.64 (0.42-0.97)<br>1.29 (0.58-2.89)	 <br><br>**0.036**<br>0.530	 <br><br>0.267<br>0.797
TTC28<br>rs9608696<br>TT<br>GG	 <br>329<br>0	 <br>156<br>0	 <br>-	 <br>-		 <br>-	 <br>-	
CDC25C<br>rs3734166<br>GG<br>GA<br>AA	 <br>185<br>133<br>20	 <br>77<br>76<br>8	 <br>Ref<br>1.69 (0.99-2.87)<br>1.59 (0.42-6.01)	 <br><br>0.053<br>0.490	 <br><br>0.342<br>0.701	 <br>Ref<br>1.70 (0.99-2.92)<br>2.20 (0.60-8.07)	 <br><br>0.056<br>0.230	 <br><br>0.267<br>0.513
GSTM1<br>+ (Present)<br>- (Null)	 275<br>55	 129<br>30	 Ref<br>1.69 (1.03-2.75)	 <br>**0.036**	 <br>0.522	 Ref<br>1.52 (0.93-2.50)	 <br>0.096	 <br>0.279

GSTT1<br>+ (Present)<br>- (Null)	 256<br>74	 126<br>33	 Ref<br>0.76 (0.49-1.20)	 <br>0.250	 <br>0.494	 Ref<br>0.75 (0.47-1.18)	 <br>0.210	 <br>0.513
*Adjusted for gender #Corrected for multiple testing using the Benjamin-Hochberg method.								
