# Supplementary material for: Influence of Genetic Polymorphisms on the Age at Cancer Diagnosis in a Homogenous Lynch Syndrome Cohort of Individuals Carrying the MLH1:c.1528C>T South African Founder Variant
Source: Biomedicines. 2024 Sep 27;12(10):2201. doi: 10.3390/biomedicines12102201 (PMC11505229; doi:10.3390/biomedicines12102201)
Supplement: Supplementary file 1 [file biomedicines-12-02201-s001.zip › Supplementary Table S6.pdf]

Supplementary Table S6. Comparison of Kaplan-Meier survival by genotype, univariate and sex-adjusted (as confounder) Cox regression analysis by genotype for CRC. Note: Significant polymorphism genotypes are in Bold. Abbreviations: HR. Hazards Ratio, CI. Confidence Interval, Ref. Reference genotype.

Polymorphism	Genotype (N)	Cancer events	Log-rank test P	Univariate HR (95% CI)	P-value	*Adjusted HR (95% CI)	P-value
HFE H63D rs1799945							
CC	249	134	0.900	Ref		Ref	
CG	50	26		0.89 (0.53-1.50)	0.670	1.00 (0.59-1.70)	0.990
CT	2	1		0.64 (0.09-4.76)	0.670	0.47 (0.06-3.47)	0.460
GG	2	1		0.51 (0.07-3.78)	0.510	0.45 (0.06-3.33)	0.430
Any G/T (CG+CT+GG)	52	21	0.890	0.87 (0.52-1.45)	0.580	0.95 (0.56-1.60)	0.840
CYP17 rs743572							
AA	68	35	0.380	Ref		Ref	
AG	156	81		1.18 (0.74- 1.90)	0.490	1.18 (0.73- 1.91)	0.500
GG	60	24		1.06 (0.56- 2.01)	0.850	1.14 (0.60- 2.18)	0.690
GT	23	13		1.29 (0.57- 2.88)	0.540	1.45 (0.64-3.29)	0.370
AT	32	10		0.51 (0.20-1.27)	0.150	0.48 (0.19- 1.21)	0.120
TT	1	0		0.000 (0.00-inf)	1.000	0.000 (0.00-inf)	1.000
Any G/T (AG+GG+GT+AT+TT)	248	104	0.580	1.09 (0.69-1.71)	0.730	1.01 (0.70-1.74)	0.680
hTERT rs2075786							
AA	98	48	0.570	Ref		Ref	
AG	143	86		1.24 (0.82-1.88)	0.310	1.18 (0.78-1.79)	0.430
GG	64	33		1.43 (0.85-2.39)	0.180	1.31 (0.78- 2.21)	0.310
Any G (AG+GG)	207	91	0.360	1.29 (0.87-1.91)	0.210	1.21 (0.82-1.80)	0.340
PPP2R2B rs10477307							
GG	116	70	0.280	Ref		Ref	
GA	148	72		0.76 (0.52- 1.15)	0.160	0.73 (0.50-1.08)	0.110
AA	40	20		0.85 (0.48- 1.56)	0.590	0.85 (0.48-1.54)	0.600
Any A (GA+AA)	188	71	0.140	0.78 (0.54-1.12)	0.180	0.75 (0.53-1.09)	0.130
KIF20A rs10038448							
CC	195	99	0.480	Ref		Ref	
GC	98	57		1.21 (0.82- 1.77)	0.340	1.31 (0.88- 1.94)	0.180
GG	13	7		1.17 (0.47-2.94)	0.740	1.33 (0.53- 3.38)	0.540
Any C (GC+GG)	111	48	0.380	1.20 (0.83-1.75)	0.340	1.31 (0.90-1.92)	0.160
TGFB1/CCDC97 rs12980942							
GG	259	145	0.290	Ref		Ref	
GA	42	15		0.56 (0.30-1.08)	0.083	0.59 (0.30-1.13)	0.110

AA	4	3		1.39 (0.41-4.74)	0.600	1.06 (0.30-3.71)	0.930
Any A (GA+AA)	46	14	0.110	0.64 (0.36-1.16)	0.140	0.65 (0.36-1.17)	0.150
XRCC5 rs1051685							
AA	163	90	**0.040**	Ref		Ref	
AG	112	58		0.59 (0.40-0.89)	**0.012**	0.61 (0.41- 0.92)	**0.019**
GG	31	15		0.79 (0.43-1.46)	0.460	0.93 (0.51-1.71)	0.830
Any G (AG+GG)	143	52	**0.019**	0.64 (0.44-0.92)	**0.017**	0.67 (0.46-0.98)	**0.038**
TNF rs3093662							
AA	243	136	0.260	Ref		Ref	
AG	58	26		1.06 (0.64- 1.75)	0.820	0.96 (0.58- 1.59)	0.880
GG	4	1		0.22 (0.03- 1.62)	0.140	0.24 (0.03- 1.72)	0.150
Any G (AG+GG)	62	23	0.480	0.89 (0.55-1.45)	0.650	0.84 (0.52-1.36)	0.480
BCL2							
rs1531697	159	76	0.560	Ref		Ref	
TT	117	72		1.11 (0.76-1.61)	0.590	1.06 (0.72- 1.54)	0.780
TA	28	14		0.98 (0.50-1.96)	0.970	0.73 (0.36- 1.47)	0.380
AA	145	61	0.840	1.09 (0.76-1.56)	0.640	0.99 (0.69-1.43)	0.970
Any A (TA+AA)							
CHFR rs11610954							
CC	260	137	0.270	Ref		Ref	
CT	40	23		1.55 (0.93- 2.60)	0.095	1.54 (0.92-2.59)	0.100
TT	6	3		1.35 (0.41- 4.44)	0.620	1.26 (0.38-4.10)	0.710
Any T (CT+TT)	46	23	0.120	1.53 (0.93-2.50)	0.094	1.50 (0.91-2.47)	0.110
CDC25C rs6874130							
GG	96	48	0.880	Ref		Ref	
GC	162	88		1.02 (0.68- 1.53)	0.910	1.12 (0.74- 1.68)	0.600
CC	48	27		1.08 (0.63- 1.85)	0.770	1.41 (0.80- 2.45)	0.230
Any C (GC+CC)	210	89	0.840	1.04 (0.71-1.53)	0.850	1.17 (0.79-1.74)	0.440
ATM							
rs1800057	300	155	0.520	Ref		Ref	
CC	6	8		1.99 (0.68-5.85)	0.210	1.63 (0.55- 4.87)	0.380
CG							
CYP1A1 Msp1 rs4646903							
AA	204	112	**0.007**	Ref		Ref	
AG	78	40		0.68 (0.44-1.04)	0.072	0.65 (0.45- 1.00)	**0.051**
GG	18	10		1.66 (0.81-3.43)	0.170	1.77 (0.85- 3.66)	0.130
Any G (AG+GG)	96	42	0.310	0.77 (0.52-1.15)	0.200	0.76 (0.51-1.13)	0.170
TTC28							
rs9608696	329	156	-	-	-	-	-
TT	0	0					

GG							
CDC25C rs3734166							
GG	171	77	0.110	Ref		Ref	
GA	115	76		1.41 (0.97- 2.05)	0.075	1.50 (1.02- 2.21)	**0.039**
AA	18	8		1.78 (0.75- 4.23)	0.190	2.27 (0.97- 5.43)	0.066
Any A (GA+AA)	133	64	0.056	1.44 (0.99-2.08)	0.053	1.55 (1.06-2.26)	**0.022**
GSTM1							
+ (Present)	249	129	0.056	Ref		Ref	
- (Null)	50	30		1.56 (0.98- 2.49)	0.061	1.52 (0.94- 2.49)	0.087
GSTT1							
+ (Present)	232	126	0.130	Ref		Ref	
- (Null)	67	33		0.62 (0.40- 0.99)	**0.044**	0.63 (0.40-1.00)	**0.048**

*Adjusted for sex
