# Supplementary material for: Influence of Genetic Polymorphisms on the Age at Cancer Diagnosis in a Homogenous Lynch Syndrome Cohort of Individuals Carrying the MLH1:c.1528C>T South African Founder Variant
Source: Biomedicines. 2024 Sep 27;12(10):2201. doi: 10.3390/biomedicines12102201 (PMC11505229; doi:10.3390/biomedicines12102201)
Supplement: Supplementary file 1 [file biomedicines-12-02201-s001.zip › Supplementary Table S7.pdf]

**Supplementary Table S7.** Multivariate and sex-adjusted (as confounder) Cox regression analysis by genotype for CRC. Note: Significant polymorphism genotypes are in Bold. Abbreviations: HR. Hazards Ratio, CI. Confidence Interval, Ref. Reference genotype.

| Polymorphism | Genotype (N) | Cancer events (N) | HR (95% CI) | P-value | #P-value | *Adjusted HR (95% CI) | P-value | #P-value |
|---|---|---|---|---|---|---|---|---|
| HFE H63D rs1799945 | | | | | | | | |
| CC | 249 | 134 | Ref | | | Ref | | |
| CG | 50 | 26 | 0.84 (0.47-1.49) | 0.550 | 0.699 | 0.86 (0.48-1.54) | 0.620 | 0.863 |
| CT | 2 | 1 | 1.15 (0.14-9.46) | 0.900 | 0.926 | 0.82 (0.10-6.68) | 0.850 | 0.968 |
| GG | 2 | 1 | 0.32 (0.04-2.50) | 0.280 | 0.613 | 0.22 (0.03-1.82) | 0.160 | 0.434 |
| CYP17 rs743572 | | | | | | | | |
| AA | 68 | 35 | Ref | | | Ref | | |
| AG | 156 | 81 | 1.74 (0.99-3.03) | 0.052 | 0.370 | 1.84 (1.05-3.22) | **0.034** | 0.234 |
| GG | 60 | 24 | 1.21 (0.60-2.44) | 0.590 | 0.699 | 1.24 (0.61-2.51) | 0.250 | 0.802 |
| GT | 23 | 13 | 1.56 (0.63-3.86) | 0.340 | 0.671 | 1.75 (0.70-4.42) | 0.230 | 0.546 |
| AT | 32 | 10 | 0.57 (0.22-1.49) | 0.250 | 0.607 | 0.57 (0.22-1.49) | 0.250 | 0.546 |
| TT | 1 | 0 | 0.000 (0.00-inf) | 1.000 | 0.999 | 0.000 (0.00-inf) | 1.000 | 0.999 |
| hTERT rs2075786 | | | | | | | | |
| AA | 98 | 48 | Ref | | | Ref | | |
| AG | 143 | 86 | 1.19 (0.76-1.87) | 0.440 | 0.699 | 1.24 (0.78-1.97) | 0.370 | 0.685 |
| GG | 64 | 33 | 1.48 (0.83-2.63) | 0.180 | 0.481 | 1.51 (0.85-2.69) | 0.160 | 0.434 |
| PPP2R2B rs10477307 | | | | | | | | |
| GG | 116 | 70 | Ref | | | Ref | | |
| GA | 148 | 72 | 0.70 (0.45-1.09) | 0.120 | 0.700 | 0.67 (0.42-1.05) | 0.081 | 0.283 |
| AA | 40 | 20 | 0.69 (0.35-1.36) | 0.290 | 0.613 | 0.71 (0.36-1.37) | 0.310 | 0.629 |
| KIF20A rs10038448 | | | | | | | | |
| CC | 195 | 99 | Ref | | | Ref | | |
| GC | 98 | 57 | 0.74 (0.37-1.47) | 0.390 | 0.672 | 0.80 (0.40-1.60) | 0.520 | 0.792 |
| GG | 13 | 7 | 0.55 (0.10-2.94) | 0.490 | 0.699 | 0.55 (0.11-2.67) | 0.460 | 0.728 |
| TGFB1/CCDC97 rs12980942 | | | | | | | | |
| GG | 259 | 145 | Ref | | | Ref | | |
| GA | 42 | 15 | 0.52 (0.25-1.04) | 0.066 | 0.370 | 0.52 (0.26-1.05) | 0.067 | 0.283 |
| AA | 4 | 3 | 2.85(0.67-12.08) | 0.160 | 0.481 | 2.37 (0.57-9.90) | 0.240 | 0.546 |
| XRCC5 rs1051685 | | | | | | | | |
| AA | 163 | 90 | Ref | | | Ref | | |
| AG | 112 | 58 | 0.55 (0.35-0.88) | **0.012** | 0.197 | 0.55 (0.35-0.88) | **0.013** | 0.167 |
| GG | 31 | 15 | 0.84 (0.43-1.63) | 0.600 | 0.699 | 0.95 (0.49-1.82) | 0.870 | 0.968 |

| | | | | | | | | |
|---|---|---|---|---|---|---|---|---|
| TNF rs3093662 | | | | | | | | |
| AA | 243 | 136 | Ref | | | Ref | | |
| AG | 58 | 26 | 1.19 (0.69-2.07) | 0.530 | 0.699 | 0.96 (0.55-1.69) | 0.890 | 0.968 |
| GG | 4 | 1 | 0.47 (0.06-3.65) | 0.470 | 0.699 | 0.36 (0.05-2.80) | 0.330 | 0.635 |
| BCL2 rs1531697 | | | | | | | | |
| TT | 159 | 76 | Ref | | | Ref | | |
| TA | 117 | 72 | 1.10 (0.72-1.67) | 0.670 | 0.740 | 0.98 (0.64-1.51) | 0.930 | 0.968 |
| AA | 28 | 14 | 0.50 (0.23-1.11) | 0.087 | 0.370 | 0.37 (0.16-0.82) | **0.014** | 0.167 |
| CHFR rs11610954 | | | | | | | | |
| CC | 260 | 137 | Ref | | | Ref | | |
| CT | 40 | 23 | 1.86 (1.04-3.33) | **0.037** | 0.370 | 1.77 (0.97-3.22) | 0.062 | 0.283 |
| TT | 6 | 3 | 1.74 (0.50-6.02) | 0.380 | 0.673 | 1.63 (0.47-5.57) | 0.440 | 0.728 |
| CDC25C rs6874130 | | | | | | | | |
| GG | 96 | 48 | Ref | | | Ref | | |
| GC | 162 | 88 | 0.96 (0.58-1.59) | 0.870 | 0.925 | 0.93 (0.56-1.55) | 0.790 | 0.968 |
| CC | 48 | 27 | 0.80 (0.37-1.74) | 0.570 | 0.699 | 0.88 (0.41-1.88) | 0.740 | 0.968 |
| ATM rs1800057 | | | | | | | | |
| CC | 300 | 155 | Ref | | | Ref | | |
| CG | 6 | 8 | 1.69 (0.50-5.72) | 0.400 | 0.672 | 1.05 (0.30-3.62) | 0.940 | |
| CYP1A1 Msp1 rs4646903 | | | | | | | | |
| AA | 204 | 112 | Ref | | | Ref | | |
| AG | 78 | 40 | 0.63 (0.39-1.02) | 0.059 | 0.370 | 0.61 (0.38-0.98) | **0.040** | 0.234 |
| GG | 18 | 10 | 1.20 (0.51- 2.85) | 0.670 | 0.740 | 1.04 (0.43-2.51) | 0.930 | 0.968 |
| TTC28 rs9608696 | | | | | | | | |
| TT | 329 | 156 | - | - | | - | - | |
| GG | 0 | 0 | | | | | | |
| CDC25C rs3734166 | | | | | | | | |
| GG | 171 | 77 | Ref | | | Ref | | |
| GA | 115 | 76 | 1.72 (0.94-3.15) | 0.077 | 0.369 | 1.61 (0.86-3.01) | 0.140 | 0.434 |
| AA | 18 | 8 | 3.52(0.74-16.76) | 0.110 | 0.400 | 3.89 (0.88-17.29) | 0.074 | 0.283 |
| GSTM1 | | | | | | | | |
| + (Present) | 249 | 129 | Ref | | | Ref | | |
| - (Null) | 50 | 30 | 2.12 (1.25-3.61) | **0.005** | 0.183 | 1.86 (1.07-3.21) | **0.027** | 0.234 |
| GSTT1 | | | | | | | | |
| + (Present) | 232 | 126 | Ref | | | Ref | | |
| - (Null) | 67 | 33 | 0.86 (0.52-1.43) | 0.560 | 0.699 | 0.80 (0.48-1.34) | 0.390 | 0.690 |

*Adjusted for gender #Corrected for multiple testing using the Benjamin-Hochberg method.
